# Bias in presence-only niche models related to sampling effort and species niches: Lessons for background point selection

**DOI:** 10.1371/journal.pone.0232078

**Published:** 2020-05-20

**Authors:** Christophe Botella, Alexis Joly, Pascal Monestiez, Pierre Bonnet, François Munoz

**Affiliations:** 1 INRIA Sophia-Antipolis - ZENITH team, Montpellier, France; 2 INRAE, UMR AMAP, Montpellier, France; 3 AMAP, Univ Montpellier, CIRAD, CNRS, INRA, IRD, Montpellier, France; 4 CIRAD, UMR AMAP, Montpellier, France; 5 INRAE, BioSP, Avignon, France; 6 Université Grenoble Alpes, Laboratoire d’Ecologie Alpine, CS 40700, Grenoble, France; University of Molise, Isernia, ITALY

## Abstract

The use of naturalist mobile applications have dramatically increased during last years, and provide huge amounts of accurately geolocated species presences records. Integrating this novel type of data in species distribution models (SDMs) raises specific methodological questions. Presence-only SDM methods require background points, which should be consistent with sampling effort across the environmental space to avoid bias. A standard approach is to use uniformly distributed background points (UB). When multiple species are sampled, another approach is to use a set of occurrences from a Target-Group of species as background points (TGOB). We here investigate estimation biases when applying TGOB and UB to opportunistic naturalist occurrences. We modelled species occurrences and observation process as a thinned Poisson point process, and express asymptotic likelihoods of UB and TGOB as a divergence between environmental densities, in order to characterize biases in species niche estimation. To illustrate our results, we simulated species occurrences with different types of niche (specialist/generalist, typical/marginal), sampling effort and TG species density. We conclude that none of the methods are immune to estimation bias, although the pitfalls are different: For UB, the niche estimate fits tends towards the product of niche and sampling densities. TGOB is unaffected by heterogeneous sampling effort, and even unbiased if the cumulated density of the TG species is constant. If it is concentrated, the estimate deviates from the range of TG density. The user must select the group of species to ensure that they are jointly abundant over the broadest environmental sub-area.

## 1 Introduction

Species Distribution Models (SDM) ([1]) based on presence-only data are widely used to characterize the ecological niches and distributions of animal and plant species across environments and space, for ecological studies and conservation planning. Popular examples of such methods include ENFA ([2]), GARP ([3]), Maxent ([4]) and more recently Bayesian methods ([5, 6]). Large amounts of presence-only data have become available through the digitization of herbarium collections ([7, 8]) and the development of citizen science, and they should improve estimation accuracy in SDM. However, sampling effort is heterogeneous and often depends on environment, yielding estimation biases in SDM ([9]). These biases are not alleviated when increasing occurrence data and require the development of methods acknowledging sampling heterogeneity.

**Fig 1:**
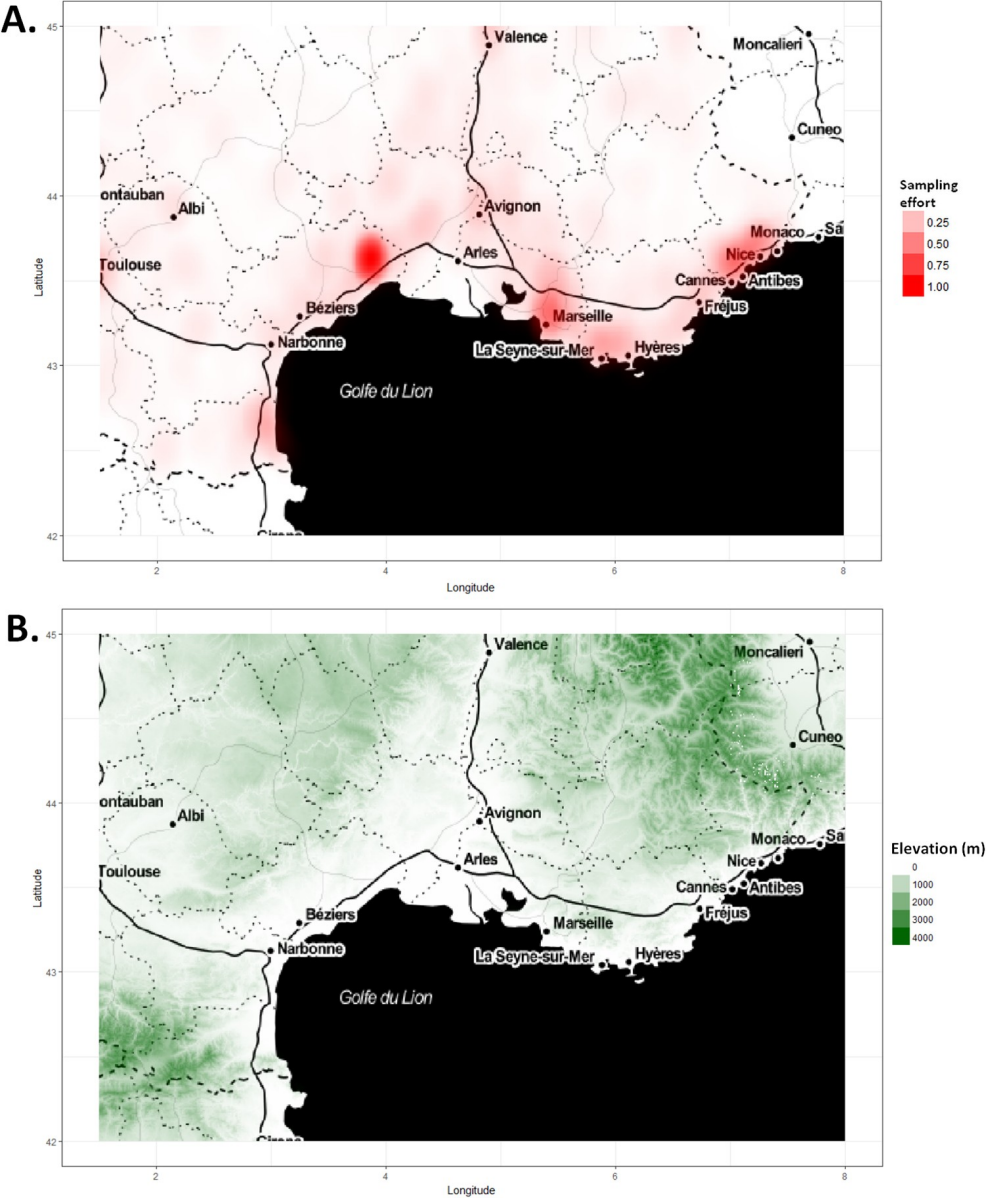
Elevation versus sampling effort in the French mediterranean region. A. An illustration of what might look like the sampling probability (or sampling effort function) over the French mediterranean region. This function is based on a kernel density estimate fitted on all the plant identifications queries sent to the Pl@ntNet mobile application system during 2016 and 2017. B. Ground elevation in meters over the French Mediterranean region. This data is extracted from the SRTM 2010 elevation database with resolution 3 arc-seconds (≈ 90 meters), see the U.S. Geological Survey website (https://lta.cr.usgs.gov/SRTMVF).

While first presence-only SDM methods like BIOCLIM ([10]) and DOMAIN ([11]) aimed at computing environmental ranges where the species could live, recent methods ([12]) look for more accuracy, and estimate the species density across environment. This density is proportional to the species expected abundance regarding only the environment. To estimate this species environmental density, such methods use a set of “background” or “pseudo-absences” points (or “quadrature” points in literature on Poisson process models, see [12], which should reflect the sampling intensity across the environmental space. Background points are usually drawn uniformly over the region, assuming a uniform sampling of the focal species distribution (default option in Maxent). However, this assumption is inadequate in most cases. Indeed, the occurrences are mostly collected without a strict sampling protocol. People visit more certain places than others, e.g. because they are closer from where they live, easier to access, biologically interesting, or aesthetically attractive. This geographic bias translates into an environmental bias, i.e. the global sampling effort that is induced by the sum of observers covaries with the environment. For instance, Fig 1 shows the that distribution of opportunistic observations of the mobile app Pl@ntNet in 2017 ([13]) is higher in lower-elevation areas. For a species specialized to mountain ecosystems, small populations at lower elevation could be over-sampled. When inferring an SDM with a uniform background, species occupancy at higher elevation would be under-estimated and the estimated niche would thus be biased toward lower elevation.

Presence-only data has evolved in availability and format. Indeed, thanks to large scale citizen-sciences programs like iNaturalist (https://www.inaturalist.org/), eBird (https://ebird.org/home), Pl@ntNet (https://plantnet.org/) or Naturgucker (https://www.naturgucker.de/), spreading the use of smartphone applications for reporting naturalist observations ([14]), presence-only data become massive in developed countries and geolocation of individual specimens becomes more accurate. In the past, most presence only data came from experts collections: Natural museums, naturalist surveys, conservatories data or environmental agencies. Observations of species presences were often aggregated to a prospection site geolocation, which spatial coverage is unknown and varies between sites. The Target-Group Background method (TGB) was proposed by [15] to correct for sampling bias in presence-only niche models in this context. It proposes to define background points as the sites where there has been at least one presence among a Target-Group of species. Today, almost each species presence reported from a mobile phone has its own geolocation and to aggregate them a posteriori in sites asks specific methodological questions. A simpler, and slightly different method is to integrate all species occurrences from the Target-Group as background. Of course, this procedure has strong links with the original TGB approach, but while TGB requires sampling effort to be homogeneous between sites to work properly, as noticed by [16] (page 429), the other method might better correct for a varying sampling effort because the concentration of occurrences from all TG species sounds more proportional to the prospection pressure in the area.

In this study, we propose a new theoretical investigation of specific advantages and biases of this approach, that we will call Target-Group Occurrences Background (TGOB) in the following. A basic problem is that the density of occurrences in the TG might be a poor approximation of the real sampling effort, because it does not only reflect sampling effort but also the varying species densities and ecological preferences of species in the TG. Thus, using Target-Group occurrences background may entail new estimation biases in SDM. However, there is no comprehensive perspective on the conditions leading to such bias. Here we address which properties of sampling effort and which ecological characteristics of species in TG can entail biases in (i) an analysis with uniform background points, and (ii) an analysis with Target-Group occurrences background.

Poisson process are useful models for presence-only SDM because they enable a clear probabilistic model and inference procedure for estimating the species environmental density. We consider Poisson process models with log-linear intensity function, which includes the most popular Maxent model ([17]). Starting from a model of species occurrences based on a thinned Poisson process where the thinning intensity is heterogeneous in space and represents the sampling effort, we first exhibited the induced Poisson process in the environmental space and showed how its intensity factorizes into the species intensity and the sampling effort averaged over space for any environment. We then re-expressed the expected density estimator as a divergence depending on focal species density, TG species density and observation density. We assessed how estimation biases arise when these densities are environmentally heterogeneous. We simulated basic cases where estimation biases are expected, for different types of sampling effort, varying niche types of the focal species (specialist vs generalist, typical vs marginal optimum), and three levels of niche breadth in TG species. We show that using background points drawn from the sampling effort proportional density is asymptotically unbiased, and show two types of bias related to alternative ways of defining background points: (i) a bias due to a mismatch of background points with actual sampling effort in the uniform background selection scheme, (ii) a bias due to ecological preferences of TG species, but irrespective of sampling heterogeneity, in TGOB.

To our knowledge, this is the first study bringing such theoretical insights to characterize sampling-related biases in presence-only SDM. Our results should help SDM users anticipate those biases, and decide whether they can use uniform, TGO backgrounds, or orientate them towards other methods and complementary data. Guidelines are provided for building the TG. It should guide good practices for performing more reliable presence only habitat models.

In **section 2**, the model of species distribution and observation is described, we introduce the form of the point process intensity in the environmental space and the observation intensity factor. In section 3, the simulation and inference settings are described. In section 4, detailed results are provided and finally, in section 5, they are discussed in order to provide guidelines for modelers.

## 2 Model of species observations

We introduce here a probabilistic model controlling the random generation of species located occurrences. It is a two step process where (i) species individuals locations are distributed according to a Poisson point process (see section 2.2), (ii) the individuals are partially observed through a random thinning operation (section 2.3). Section 2.3 also introduces an intermediary result, showing how the expected density of occurrences in the environmental space factorizes with an observation density factor that will be crucial to determine the bias of species density estimation. Before anything else, section 2.1 introduces some notations used all along the article, and the reader may find all notations are summarized and explained in Table 1.

**Table 1:** Notations summary: Mathematical notation, name, definition and meaning in our model. *Almost everywhere.

Notation	Name	Formal definition	Role in model
*D*	Geographic domain	$D\subset R^{2}$ bounded	Represent the study area
*x*	Environmental variable	D→R continuous a.e.* and bounded	Enviro. variable measured over *D* ex: anual precipitations
λ	Species intensity	$\lambda :R\to R^{+}$ continuous a.e.* and bounded on any bounded subset	Expected species abundance per space unit
*f*	Species density	$f:R\to R^{+}$, $f≔\frac{\lambda }{\int_{R}\lambda d\mu }$	Density derived from λ over R
*s*	Sampling effort	*s*: *D* → [0, 1] continuous	Locally represents the probability to report a species individual
$\bar{s}$	Observation intensity	$\bar{s}:R\to \left[0,1\right]$, Expressed in Eq 1	Avg. sampling effort on areas of *D* where *x* = *w*
*s*_*x*_	Observation density	$s_{x}:R\to R^{+}$, $s_{x}≔\frac{\bar{s}}{\int_{R}\bar{s}d\mu }$	Density derived from $\bar{s}$ over R. Controls UB bias, see Eq 2
*a*	Cumulated Target-Group species density	$a:R\to R^{+}$, $a≔\frac{\sum_{i=1}^{N}\lambda^{i}}{\int_{R}\left(\sum_{i=1}^{N}\lambda^{i}\right)d\mu }$	Controls TGOB bias see Eq 5

### 2.1 Notations

We define a measured two dimensional space (D,L(D),μ), where L(D) is the Lebesgue *σ*-algebra over *D*, a bounded subset of $R^{2}$, and *μ* is the Lebesgue measure on $R^{2}$, which can be understood as the standard measure of area. Individuals of a species are represented by points distributed over *D*, and only a part of them is reported by observers. Over this domain we consider an environmental variable that is represented by a measurable function x:D→R, continuous almost everywhere and bounded. We note Im(x)={w∈R,∃z∈D,xiscontinuousatzandx(z)=w}. Then, ∀W⊂R, we note *x*^−1^(*W*) = {*z* ∈ *D*, *x*(*z*) ∈ *W*}. We deal here with a single environmental variable *x* for clarity, but the results can be extended to more variables with the same method. We also define *μ*_*x*_, the geographic area where *x* takes a certain range of values: For all subset of environment value $W\in L\left(R\right),\;\mu_{x}\left(W\right)=\mu \left\{x^{-1}\left(W\right)\right\}=\int_{x^{-1}\left(W\right)}1d\mu$, where L(R) is the Lebesgue *σ*-algebra over R. The almost continuity of *x* means that *μ*_x_(Im(*x*)) = *μ*(*D*), i.e. the spatial area over which *x* is continuous equals the area of *D*, or said differently, the area of all points of discontinuity of *x* taken together is null. This hypothesis allows us to deal either with a continuously varying variable (e.g. defined by a mathematical function over space), or a locally discontinuous one, typically like raster environmental data (see for example [18] for a review on commonly used environmental variables in plants SDM), and even a mixture of both. For example, *x* could be the elevation variable illustrated by Fig 1. Thus, this hypothesis makes our analysis quite general regarding *x*.

### 2.2 Distribution model

Species individuals are represented by the random set *Z* of their positions in *D*. We assume *Z* is distributed according to an inhomogeneous Poisson process over *D* with intensity function $\lambda \text{o}x:D\to R^{+}$, where o is functions composition. The intensity λ depends on the environmental variable *x*. We assume it is continuous almost everywhere on R, has bounded values on any bounded subset of R and note: *Z* ∼ *IPP*(λo*x*(.)). Poisson process have indeed been proposed and used as natural probabilistic models for the distribution of species individuals in space ([12, 16]). The intensity represents the punctual limit of the expected species abundance per space unit. We note, $\forall w\in R,\;f\left(w\right)=\frac{\lambda (w)}{\int_{R}\lambda \left(u\right)du}$, a formal definition of the ecological concept of the species response function to variable *x* ([19, 20]). It can be seen as the probability density function of the random environmental variable *x*(*z*) of any individual random location *z* inside a virtual geographic space where all possible environmental values of *x* are equally represented in terms of area (this is not necessarily the case in *D*). In short, we call *f* the species density. The inhomogeneous Poisson process model proposed here represents a broad class of presence-only SDM including the popular Maxent model, even though Maxent further uses a L1 penalty for model selection. This regularization was not integrated in the study as it doesn’t change the incidence of sampling bias.

### 2.3 Observation model and observation density along the environmental gradient

We use a probabilistic model of observation in order to study the effect of heterogeneous sampling effort on bias. It is similar to the models used in [4, 15, 16, 21]. We consider a continuous **sampling effort** function *s*: *D* → [0, 1]. For any point *z* ∈ *D* where an individual of some species is located, the probability to report it is *s*(*z*). Note that *s* is not a probability density over *D*. There is, of course, no occurrences apart from true locations of individuals. Under this model, the thinning property of inhomogeneous Poisson process ([22]), called Prekopa’s theorem, states that reported presences of the species *Z*_*r*_ are distributed according to *Z*_*r*_ ∼ *IPP*(*s*(.)λ ∘ *x*(.)). To understand more clearly sampling bias on estimated niche, we propose to look rather at the environmental space rather than the geographic space. Indeed, we are especially interested in the bias of the estimated species density, which is a function of the environmental variables. However, estimation bias will depend on the sampling effort, which is defined over the geographic space but may be transposed to the environmental space. Our first and intermediary result (proved in Text A of S1 Appendix) is that the distribution of the observed species individuals in the environmental space R also follows a general Poisson process ([22, 23]) whose measure is, for any W∈R, $\int_{W}\lambda \bar{s}d\mu_{x}$ and intensity $\lambda \bar{s}$. Where $\bar{s}$ is defined by Eq 1. This intensity function $\lambda \left(w\right)\bar{s}\left(w\right)$ in environment *w* represents the expected number of occurrences on any spatial unit where the environment is constant and equal to *w*, given the underlying shape of the sampling effort *s*. We show that it is the product of the species intensity λ and the average of the sampling effort $\bar{s}$ across all areas of *D* with the given environment. This factorization appears because the species intensity is a function of *x*.

$$\begin{array}{c} \forall w\in R,\bar{s}\left(w\right)=\{\begin{array}{cc} \underset{\delta \to 0}{\text{lim}}\frac{\int_{x^{-1}\left(\left[w-\frac{\delta }{2},w+\frac{\delta }{2}\right]\right)}sd\mu }{\mu_{x}\left(\left[w-\frac{\delta }{2},w+\frac{\delta }{2}\right]\right))} & \text{if}\;w\in \text{Im}(x)\\ 0 & \text{otherwise,}\;\text{by}\;\text{convention}. \end{array} \end{array}$$ (1)

We note *s*_*x*_ the environmental density associated to $\bar{s}$ on R, called the **observation density**: $\forall w\in R,\;\;s_{x}\left(w\right)=\frac{\bar{s}\left(w\right)}{\int_{R}\bar{s}d\mu }$. In other words, *s*_*x*_ is the probability density of *x*(*z*) when *z* is randomly drawn over *D* according to the proportional density of the sampling effort (*s*/∫_*D*_ *sdμ*). For example, if the environment where observers spend the most time per area unit is *x* = *w*, then *s*_*x*_(*w*) will be the maximum of *s*_*x*_. The results section will tell precisely how *s*_*x*_ induce bias with the uniform background scheme.

## 3 Simulation and inference setting

**Fig 2:**
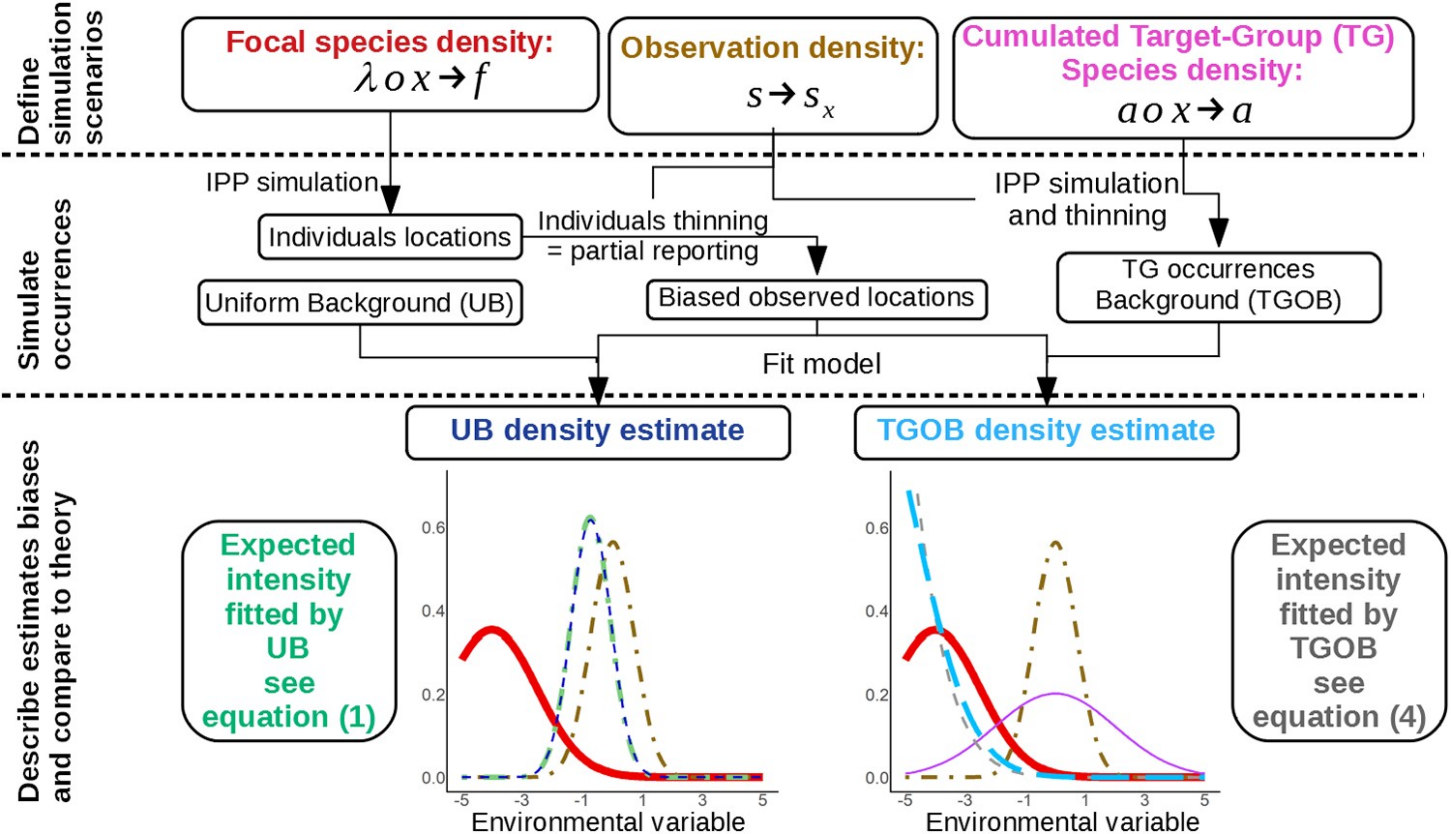
Illustration of the simulation experiment procedure used in this paper to evaluate species density estimation bias under various scenarios. This flowchart shows the role of every component (i.e. the focal species intensity *f*, the observation density *s*_*x*_, and the cumulated TG species density *a*) in the simulation of occurrences, the density estimation with TGOB and UB, and the illustrative comparison of the estimates with the theoretical expectations respectively exhibited by Eqs 2 and 5.

To clarify and illustrate the practical consequences of the mathematical results presented in section **4**, we carry out a simulation experiment exhibiting the estimation biases in various scenarios. In the following, **UB** denotes the estimation of a Poisson Point Process model with uniform background, and **TGOB** the Target-Group occurrences background alternative. We simulate large samples of observed points of a focal species under contrasted scenarios of focal species density and observation density shapes. We also generate a large set of alternatively uniform or Target-Group background points, with various shapes of species cumulated density for the latter. We carry out the species density model estimation from the given focal species observed points and background points. We finally plot the estimated density, approximating the expected estimation, against the true one and the observation density along the enviromnental variable axis. For UB, we also plot the focal species occurrences, that is the theoretically expected density estimate, while for TGOB we plot the TG species cumulated density shape and the theoretically expected density estimate. This experimental procedure is summarized in diagram of Fig 2. This part presents each step of the simulation scheme and technical settings.

### 3.1 Environmental variable

We consider a square spatial domain *D* = [−5, 5]^2^ where the environmental variable *x* is a linear gradient from west to east, such that *x*(*z*) = *z*_1_. In this setting, *μ*_*x*_ is equal to the restriction of the R-Lebesgue measure to Im(*x*) = [−5, 5], *i.e*. each *x* value has the same spatial extent, and thus the estimate will not be better in most represented values. Illustrations of the density derived from *μ*_*x*_, Im(*x*), an observation density and species density (see further) are provided in S1 Fig.

### 3.2 Focal species

**Fig 3:**
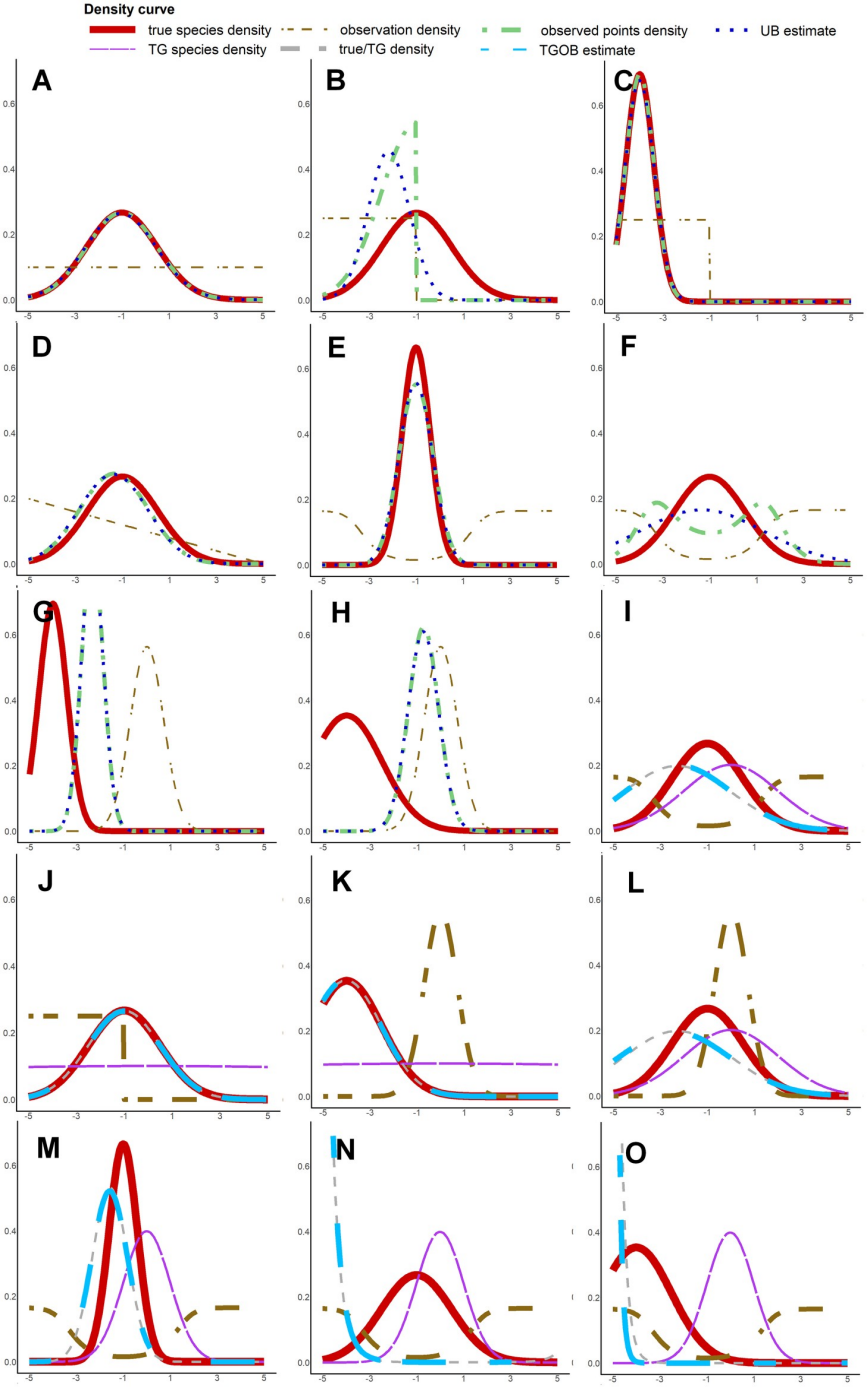
Plot of the estimated niche density with UB (A-H) and TGOB (I-O) methods for a selection of simulation situations. The different curves are: The focal species intensity function (*f*), observation density (*s*_*x*_), observed points density (λ_0_ *s*_*x*_, in UB graphs), Target-Group species density (*a*, in TGOB graphs), ratio density of species over target group (λ_0_/*a*, in TGOB graphs), UB and TGOB estimators of species density from simulated points. A-*μ*_0_ = −1; *σ*_0_ = 1.5; *obs* = *CST*. B-*μ*_0_ = −1; *σ*_0_ = 1.5; *obs* = *CUT*. C-*μ*_0_ = −4; *σ*_0_ = 0.6; *obs* = *CUT*. D-*μ*_0_ = −1; *σ*_0_ = 1.5; *obs* = *LIN*. E-*μ*_0_ = −1; *σ*_0_ = 0.6; *obs* = *HOL*. F-*μ*_0_ = −1; *σ*_0_ = 1.5; *obs* = *HOL*. G-*μ*_0_ = −4; *σ*_0_ = 0.6; *obs* = *GS*. H-*μ*_0_ = −4; *σ*_0_ = 1.5; *obs* = *GS*. I-*μ*_0_ = −1; *σ*_0_ = 1.5; *obs* = *HOL*. J-*μ*_0_ = −1; *σ*_0_ = 1.5; *obs* = *CUT*. K-*μ*_0_ = −4; *σ*_0_ = 1.5; *obs* = *GS*. L-*μ*_0_ = −1; *σ*_0_ = 1.5; *obs* = *GS*. M-*μ*_0_ = −1; *σ*_0_ = 0.6; *obs* = *HOL*. N-*μ*_0_ = −1; *σ*_0_ = 1.5; *obs* = *HOL*. A-*μ*_0_ = −4; *σ*_0_ = 0.6; *obs* = *HOL*.

The species density *f* is the probability density function of the environmental value of a specimen random location. We model it with a Gaussian function, which is a standard assumption related to the representation of species distribution over environmental gradients ([19, 24]). We give some insights about the underlying model assumptions in **Text B of** S1 Appendix. The mean of *f* is called *μ*_0_, it is the environmental optimum of the species, and we take *μ*_0_ ∈ {−1, −4} (typical vs marginal). Besides, *σ*_0_ is the standard deviation, or the niche breadth of the species, and we take *σ*_0_ ∈ {0.6, 1.5}, for a specialist or generalist species. We thus simulate 4 virtual species. *f* is illustrated in each graph of Fig 3.

### 3.3 Types of observation density

We want to estimate the density of the focal species from reported points. We examine how the bias in estimated intensity is related to *s*_*x*_, the observation density in Im(*x*). We define several shapes for *s*_*x*_ in Im(*x*), which is illustrated with the yellow curve in each graph of Fig 3:

1. Constant (CST), representing unbiased, constant observation over the domain. See graph A.
2. (1/10) − (*x*/50), *i.e*. linearly decreasing from west to east (LIN). See graph D.
3. $\frac{1_{x\in [-5,0]}}{5}$, constant observation on the lower part of the domain (CUT). See graph B.
4. $\frac{\text{log}(1+{\left(x+1\right)}^{2})}{\int_{\left[-5,5\right]}\text{log}\left(1+{\left(w+1\right)}^{2}\right)dw}$, with depleted observation density around -1 (HOL). See graph E.
5. A standard normal distribution (NOR). See graph G.

Note that *s*_*x*_ is determined through the definition of the sampling effort *s* which is in the spatial domain. We set the sampling effort to be constant along the second dimension of space (latitude) in our simulation setting, which enforces *s*_*x*_ *αs* and we thus control the shape of *s*_*w*_ through the shape of *s* over the longitude.

### 3.4 Target group of species

TGOB method uses occurrences from a set of species called the Target Group (TG) as background points in the inference setting (see methods implementation below). We thus simulate the TG occurrences background by generating occurrences of *N* independent species, constituting the TG, through their observed intensities. For species *i*, its local observed intensity takes values $\lambda^{i}\left(x\left(z\right)\right)\bar{s}\left(x\left(z\right)\right),\forall z\in D$ (assuming constant detection in space), and regrouping occurrences of all TG species is equivalent to drawing points with a global intensity $C_{a}a\left(x\left(z\right)\right)\bar{s}\left(x\left(z\right)\right)=\sum_{i=1}^{N}\lambda^{i}\left(x\left(z\right)\right)\bar{s}\left(x\left(z\right)\right)$, where $a\left(x\left(z\right)\right)≔\sum_{i=1}^{N}\lambda^{i}\left(x\left(z\right)\right)/C_{a}$ is called the TG species cumulated density and $C_{a}≔=\int_{R}\left(\sum_{i=1}^{N}\lambda^{i}\right)d\mu$ is its normalisation constant. As it is shown further, *a* will determine the bias of TGOB. Thus, we do not define each TG species density individually in the simulation, but rather test 3 shapes of *a*. It enables to visualize clearly its effect on TGOB bias: (i) FLAT: A Gaussian density of mean 0 and standard deviation 20 (≈ constant), (ii) THICK: A Gaussian density of mean 0 and standard deviation 2 and (iii) THIN: A Gaussian density of mean 0 and standard deviation 1. They are represented in, respectively, graphs J, I and M of Fig 3.

### 3.5 Simulating observation points

Statistical theory insures that the density estimate will converge towards its expectation when increasing the size of the sample. Then, for all simulations, we generate a very large sample of points (occurrences and background) so that the estimate approximates well this expectation, insuring that the estimation error is completely due to bias and not the randomness of the sample. To generate points according to a Poisson process of intensity function *f* on Im(*x*), we first determine an upper bound *B* of *f* on Im(*x*). Then, we repeat (i) Draw a point *z* ∼ *U*(*D*), (ii) Draw a variable *y* ∼ *U*([0, *B*]), (iii) We accept *z* if *y* <= *f*(*x*(*z*)) and (iv) If 20000 points are accepted, finish the procedure, otherwise go back to (i). This algorithm is applied to the focal species observed points, target group observed points and background points (see next section). 20000 points were enough for convergence of all estimates in UB and TGOB.

### 3.6 Computation of models and software

In the UB method, we estimate the model parameters with the standard maximum likelihood approach. We use the Poisson process likelihood approximation of [25], which transform the original likelihood to a Poisson regression likelihood, using background points. We draw the background points uniformly in the spatial domain *D*. Details on the construction of approximation, the weighting of points and the reparametrization of *μ*_0_ and *σ*_0_ are presented in **Text C of** S1 Appendix. As the objective function is a particular case of Generalized Linear Model likelihood, we fit the parameters using the standard R package **glm**. For *TGOB* method, the procedure is the same except that the background points are independently drawn from the density *sa*/∫_*D*_ *sadμ* rather than uniformly on *D*.

## 4 Results

We present results on estimation biases for UB and TGOB methods based on both a mathematical analysis and simulation. Our main results are formal Eqs (2) and (5) which express the target of the density estimate in the environmental space as a function of the true focal species density *f*, the observation density *s*_*x*_ (for UB) or the cumulated TG species density *a* (for TGOB) given the generative model described in section **2**. Estimation bias then depends on the instanciation of *f* and *s*_*x*_ for UB, or of *f* and *a* for TGOB. We qualitatively describe the bias, i.e. the estimated density deviation compared to the true one, that will appear depending on the shape of the dependent densities: The observation density (for UB in sections **4.2, 4.4, 4.5, 4.6** and for TGOB in **4.8**), the focal species density (for UB in **4.3**, and for TGOB in **4.9, 4.10**) and the Target-Group species density for TGOB (**4.8, 4.9**). This qualitative description are based on interpretation of Eqs 2, 3, 4, 5 and 6. This qualitative description of bias is numerically illustrated with several simulated scenarios. Graphs of all simulated scenarios are represented in S2 Fig for UB, and S3, S4 and S5 Figs for TGOB. R scripts for running the simulations and generating the graphs can be found in at https://github.com/ChrisBotella/UB-and-TGOB. Results are presented here for a single environmental variable. In the case of several environmental variables *x*_1_, …, *x*_*p*_, the Kullback-Leibler (KL) divergence used in the following equations is simply applied to densities over the multidimensional space, with adapted definitions for $s_{x_{1},\ldots ,x_{p}}$ and $\mu_{x_{1},\ldots ,x_{p}}$. For simplifying notations, we will possibly mean, by the notation of a function, a product or a quotient of functions, the density associated with it on its definition space, and this in all that follows. For example, *fs*_*x*_ refers to the proportional density function *fs*_*x*_/∫_Im(*x*)_ *fs*_*x*_ *dμ*_*x*_ over Im(*x*).

### 4.1 UB: Niche estimate minimizes KL divergence from observed density

We show in **Text D of** S1 Appendix that the expectation of the parameters estimates of the UB method is:

$$\begin{array}{c} E\left({\hat{\theta }}_{UB}\right)={\text{argmin}}_{\theta }\;D_{\text{KL}}^{\mu_{x}}(fs_{x}\left|\right|f_{\theta }) \end{array}$$ (2)

Eq 2 means that the estimated species density $f_{{\hat{\theta }}_{UB}}$ will fit the observed environmental density *fs*_*x*_ as close as possible within the parametrization constraints in term of the KL Divergence with measure *μ*_*x*_ (*μ*_*x*_-almost everywhere). For example, in our simulation model, $f_{{\hat{\theta }}_{UB}}$ is Gaussian, so it cannot fit perfectly to *fs*_*x*_ which is non-Gaussian (see graph **B** of Fig 3), but achieves the best Gaussian approximation. However, in the case where *s*_*x*_ and *f* are two Gaussian densities with distinct means and variances, *fs*_*x*_ will also be Gaussian [26]. Thus, $f_{{\hat{\theta }}_{UB}}$ will exactly converge to *fs*_*x*_ (see graph **H** of Fig 3). However, it has a different mean and variance from *f*, so that the UB estimate is biased. A Complementary explanation about the significance of *μ*_*x*_ for the KL-Divergence, and its consequences are given in **Text E of** S1 Appendix.

### 4.2 UB: Bias is small for small variations of observation density over the species niche

UB bias is tightly linked to the concentration of the observation density in the environmental space but this concept of concentration is hard to define. Still, as a density get less concentrated it get closer to a uniform density, and its variation get close to zero everywhere. Thus, we study the effects of variations of *s*_*x*_ and *f* on bias, we propose an explanation of the bias behavior observed in simulation through a simple analysis based on the density functions derivatives. For this purpose, both density functions are assumed to be differentiable over Im(*x*), which is true in the simulation setting, except in the case of observation type **CUT**. Eq 3 shows that when *s*_*x*_ varies little, the observed points density *s*_*x*_ *f*, which is fitted by the UB estimate, will get close to the true species density *f*.

$$\begin{array}{c} \underset{\text{max}|\partial s_{x}/\partial x|\to 0}{\text{lim}}\frac{\partial fs_{x}}{\partial x}=\underset{\text{max}|\partial s_{x}/\partial x|\to 0}{\text{lim}}\frac{\left(\frac{\partial f}{\partial x}s_{x}+\frac{\partial s_{x}}{\partial x}f\right)}{\int_{R}f\;s_{x}\;d\mu_{x}}=\frac{\partial f}{\partial x} \end{array}$$ (3)

Fig 3A confirms that UB is not biased when observation density is constant: The species true density *f* (red curve) is equal to the observed point density *s*_*x*_ *f* (green curve), which is perfectly fitted by the UB estimated density (blue curve). Even for graph D, the gap between true and estimated density is very small. This behavior is explained by Eq 3: If linearly decreasing observation density varies slowly, i.e. *max*|∂*s*_*x*_/∂*x* is close to zero, the derivative of the target $\partial f_{\theta_{0}}s_{x}\approx f_{\theta_{0}}/\partial x$ is close to the derivative of the species true density, implying that the estimate will fit this density. In addition, in environments where species specimens are rare, very low observation density doesn’t affect the global estimate. Type *CUT* illustrates this: There is almost no bias for *μ*_0_ = −4 (graph C of Fig 3), as the observed species density (green curve) is very close to the true species density (red curve). We note as a side remark that the differentiability of *s*_*x*_ over Im(*x*) is not necessary. It depends on complex conditions on *x* and *s*. As a counter example, continuity of *s*_*x*_ doesn’t even have a standard sense if *x* is defined by a geographic raster. Indeed, Im(*x*) is then discrete set of *x* values taken over the raster cells, and $\bar{s}$ is only defined on these values which don’t include any continuum of real numbers. The differentiability is only assumed here to analyse the effects of *s*_*x*_ variations in a simplified context.

### 4.3 UB: Smaller bias for more specialist species

The comparison of the graphs **G** (specialist) to **H** (generalist) in Fig 3 shows that the bias on niche optimum and breadth estimates is stronger for the generalist species. Indeed, we deduce from Eq 4 that *fs*_*x*_ approaches *s*_*x*_ as the variation of *f* over Im(*x*) decreases.

$$\begin{array}{c} \underset{\text{max}|\partial f/\partial x|\to 0}{\text{lim}}\frac{\partial fs_{x}}{\partial x}=\underset{\text{max}|\partial f/\partial x|\to 0}{\text{lim}}\frac{\left(\frac{\partial f}{\partial x}s_{x}+\frac{\partial s_{x}}{\partial x}f\right)}{\int_{R}fs_{x}d\mu_{x}}=\frac{\partial s_{x}}{\partial x} \end{array}$$ (4)

We can thus say that for a generalist species, the variation speed of *s*_*x*_ is high compared to the one of *f*, and UB estimate will fit more the observation density than the species density.

### 4.4 UB: Over-estimated specialization when sampling effort is concentrated

When the observation density is highly concentrated in a restricted range of the environment, as with the type *GS*, UB estimates that the species is more specialized than it is actually (see graphs G and H of Fig 3). The estimated niche variance is then lower than expected.

### 4.5 UB: Strong deviations from optimum

Graphs **B** and **H** in Fig 3 show that, when the observation density is concentrated far from the optimum of the species density, we get a strongly deviated estimated optimum. This might be very misleading for ecological analysis. Estimation of graph **H** suggests that the species is the most abundant in a range where it is actually cryptic.

### 4.6 UB: Sampling marginal specimens means over-estimating generalism

Graph **F** of Fig 3 shows that when the observation is more intense in the margin of the species niche, UB over-estimates the niche breadth of the species. This case represent observers having more interest in reporting a species out of its typical environment.

### 4.7 TGOB: Integrating samples from a Target Group of species

Firstly, using the same analytical approach as previously, we show in **Text F** of S1 Appendix that drawing directly background points from the sampling effort proportional density *s*/∫_*D*_ *s*(*z*)*dz* give unbiased species intensity estimate. This answers an open question of [4] who introduced this theoretical method (called ApproxFactorBiasOut in the article). Unfortunately, we rarely have directly access to a true sample from the sampling effort distribution. An interesting alternative is to use Target-Group species occurrences as background points (TGOB), i.e. making the hypothesis that those occurrences are approximately drawn from the sampling effort proportional density. We will investigate biases occurring with this method and a necessary and sufficient condition on Target-Group species to avoid them under our modeling hypothesis. In the following, we introduce an equation showing the displaced target of the TGOB estimator. It shows how the cumulated TG species density, especially when it is concentrated in restricted environments, can bias the estimated focal species density. We have a target group of *N* species whose individuals are distributed independently according to the species model described above, and reported from the same area *D* with the probability of observation *s* (same as the species of interest), giving for each of them a set of observation locations ${\left(Z^{i}\right)}_{i\in [|1,n_{tg}|]}$. $\forall i\in \left[|1,N|\right],\;Z^{i}∼IPP\left(s\;\lambda^{i}\circ x\right)$. We assume a constant detection probability of individuals across space for any species conditionally to observation. Then, the global set of Target Group observations locations is $Z^{tg}≔\underset{i\in [|1,N|]}{\cup }Z^{i}∼IPP\left(s\;a\circ x\right)$, where $\forall z\in D,a\left(x\left(z\right)\right)≔\sum_{i=1}^{N}\lambda^{i}\left(x\left(z\right)\right))$ is the cumulated TG species intensity. The expected estimate of TGOB is:

$$\begin{array}{c} E\left({\hat{\theta }}_{TGOB}\right)={\text{argmin}}_{\theta }\;D_{\text{KL}}^{\mu_{x}}(fs_{x}\left|\right|f_{\theta }s_{x}a) \end{array}$$ (5)

The proof is given in **Text G of** S1 Appendix. If ∀*w* ∈ Im(*x*), *a*(*w*) > 0, we can set *f*_*θ*_ ≔ *f*/*a* to cancel the divergence. Eq 5 means the TGOB estimate is expected to fit to density *f*/*a*, which is independent of the observation density, but depends on the cumulated TG species density. This result leads to the following consequences described in sections **4.8, 4.9** and **4.10**.

### 4.8 TGOB: If *a* is constant, TGOB is unbiased

We can see that when *a* is constant, *s*_*x*_ *a α s*_*x*_. Thus, the background points are distributed according to the sampling effort, and TGOB yields an unbiased estimation as ApproxFactorBiasOut. This is true whatever is the observation density. We illustrate it in two cases of Fig 3: *μ*_0_ = −1;*σ*_0_ = 1.5; *CUT* with graph J and *μ*_0_ = −4; *σ*_0_ = 1.5; *GS* with graph **K**. Here the TGOB estimator approaches almost perfectly the true species density, correcting well for unbalanced observation density in both cases, while in those same cases UB gives a strongly biased estimate. Furthermore, even with non constant *a*, the different types of observation density never affect TGOB. The bias is only due to the Target Group species density. For example, graphs **I** and **L** of Fig 3 show that TGOB estimator do not change in two very different observation density situations, *HOL* and *GS*, but with the same species density {*μ*_0_ = −1, *σ*_0_ = 1.5} and TG.

### 4.9 TGOB: The estimate deviates from a peaky Target Group species density

The more the Target Group species density (*a*) is concentrated in some range of *x*, the more our niche estimate will be located outside of this range. It may entail an over estimation of niche breadth, a bias in optimum, or even an hyper-concentration on the borders. To show this, we can analyse the effect of the variation speed of *a* and *f*, by again assuming that they are differentiable over Im(*x*) and examining the derivative of *f*/*a*:

$$\begin{array}{c} \frac{\partial f/a}{\partial x}=\frac{1}{a}(\frac{\partial f}{\partial x}-\frac{f}{a}\frac{\partial a}{\partial x}) \end{array}$$ (6)

If *a* gets high in a neighborhood *v* of Im(*x*), we will have *f*/*a* → 0 on *v*, and $\frac{\partial f/a}{\partial x}$ tends to 0 as well. Our estimate then becomes flat and low on *v* as it fits to *f*/*a*. In parallel, *a* is low outside of *v* because it must integrate to 1. Therefore, in Im(*x*)\*v*, we will have *f*/*a* → + ∞, and its derivative becomes important with the same sign as $-\frac{\partial a}{\partial x}$. In summary, as *a* concentrates in a neighborhood *v*, our TGOB estimate becomes flat and low on *v*, while it increases outside of *v*, with bigger slopes where *a* varies. This expulsion phenomenon entails bias in optimum and variance estimation. Thus, the magnitude of bias depends on the concentration of *a*, but also on the marginality of the optimum of the focal species (*μ*_0_) compared to the one of the Target-Group. Indeed, the graphs **I** and **M** of Fig 3 show that when the species optimum is close to the one of the TG density (typical species), the niche breadth is over-estimated. There is also a small deviation in optimum because the focal species is not centered around the TG optimum. In other words, the focal species density overlaying with the cumulated TG species density is deviated outside in the estimate. On the contrary, when the species optimum is far from the cumulated TG species density optimum (marginal species, see graph **O** of Fig 3), or when the cumulated TG density is just more concentrated (compare graph **N** to **I** in Fig 3), the situation is worse. The estimate cancels on the range of the cumulated TG species density, while it gets hyper-concentrated outside. In summary, the more the Target Group of species has a global environmental preference and the focal species is marginal, the more its niche estimate will be dispersed, or expelled, out of this environment.

### 4.1 TGOB: Stronger bias for generalist species

When comparing graph **M** to **N** in Fig 3, we see that TGOB is more biased on generalist species. For a generalist species, the estimate is more expelled from the TG species density volume. Thus, generalism of the focal species increases bias in both UB and TGOB, but the cause of bias differs, respectively, the heterogeneity of observation density and the TG global density. As UB fits the product of *f* and *s*_*x*_, TGOB does the same with the product of *f* and 1/*a*, and the latter varies in $-\frac{\partial a}{\partial x}\frac{f}{a^{2}}$ because the variation of *f* is small.

## 5 Discussion

In this study, we have explained two types of bias related to the way to define background points: the **sampling selection bias** in UB and the the **TG definition bias** in TGOB. The former case concerns the way background points reflect sampling heterogeneity, while the latter case concerns the influence of ecological preferences in TG species.

Concerning UB, our results confirm some empirical results in Maxent literature. The niche estimate will fit to the product of the focal species and observation densities. A major consequence is that bias is stronger for generalist species. Bias is also strong when the sampling effort is concentrated towards places representing a restricted range of environmental values, which happens when observers have specific preferences towards these restricted conditions. This will overestimate species specialization. Conversely, observing a species more intensively at the margin of its niche leads to overestimate niche breadth.

If the Target-Group is well selected, the method Target-Group occurrences background does account for varying sampling effort. A well selected Target-Group means that the sum of Target-Group species intensities is constant across environments. However, it is biased when this cumulated intensity of TG species varies in the environmental space, e.g. when there is some systematic environmental preference among TG species. In this case, the magnitude of bias will depend on the concentration of the TG density (depending on the TG species), the generalism of the focal species, and the marginality of its niche compared to the TG density. As the TG species density gets more concentrated compared to the focal species niche, the niche breadth will be over-estimated, and ultimately focal species density will strongly deviate from TG density. If TG species density approaches 0 faster than the species of interest in some environmental range, TGOB estimator should dramatically increase there, overriding variations elsewhere. Including the focal species in the Target-Group should partly prevent the niche expulsion effect because at least background points from the focal species will cover its niche. Also, the ecological niche of the focal species plays an important role. A generalist species is more affected by bias, as well as species with marginal niche compared to the TG density. On the contrary, when applied to a non-marginal focal species, TGOB will overestimate the niche breadth, or from another point of view, the effect of corresponding covariates will be reduced. This covariate effect cancellation will be all the stronger with Maxent ([27]) because of its Lasso regularisation. We recommend to carefully chose Target Group of species so as to insure, at least, that there are TG occurrences in the widest environmental subspace associated with the study domain. It will insure that at least one of the TG species is present in any kind of environments. Generalist species over each environmental variable should be included if possible to overall decrease the variation of the cumulated TG species density. The modeler must avoid using TGOB if presences of the focal species reach marginal environments compared to the whole Target-Group distribution.

Alternatives methods to TGOB and UB to account for sampling bias in presence only SDMs may be more suited in certain situations. [28] proposed to model sampling effort with distinct environmental variables from the species intensity (e.g. distance to roads or to cities). Thus it removes species intensity bias due to the covariation of sampling effort and species intensity covariates. However, often some covariates influence both sampling and species density. Still, our results support this approach if the sampling effort variation along its dedicated covariates is stronger than the species intensity variation (Eq 4), and the species intensity variation along its covariate is stronger than the sampling effort variation (Eq 3). Besides, for modelers who can access complementary systematic survey data, integrated models combining occurrences and presence-absence data have been developed in [16] and [29] with the same goal. In the same spirit, models combining presence-background with site-occupancy data ([30]) may be another efficient way to account for sampling bias.

We underline that our results directly concern a vast class of presence-only SDM called Poisson process models ([12]) whose intensity function is strictly positive. Indeed, modelers may use different variables transformations as predictors (GAM [31], MARS [32]), or learn those transformations automatically, like with deep neural networks ([33]). Qualitatively speaking, bias behaviors extend to L1 penalized Poisson process methods like Maxent ([34]) and to other related SDMs methods (whose predictive function is based on covariates) when using pseudo-absences, e.g. GARP ([3]), ENFA ([2]), or BRT ([1]). Models integrating interactions effects between species, called joint SDMs ([35]), should be similarly affected by described biases, as species interactions are assumed independent of the environment, but a specific investigation on biases of such methods would be important in view of the recent attention they are receiving in ecology. We notice that potential biases of the studied methods are not restricted to the ones presented here, and the modeler must be careful to other sources of errors. For example, other authors recently studied how the interaction of environmental variables resolution and niche breadth induce bias ([36]). Besides, model errors might not be due to biases, but rather to estimation variance which is also investigated in the SDM literature ([37, 38, 39]). A limitation of this study is that we did not study some other proposed sampling bias correction methods, such as occurrence thinning procedures, in spatial ([40, 41]) or environmental ([42]) domains. As occurrences thinning increases the entropy of the observed points density, it brings its own bias which should be investigated more closely. Such procedures could be studied through the formalism that we are developing.

TGOB is exactly equivalent to TGB, proposed by [15], if each TG site (defined either by the environmental rasters or the spatial aggregation of the occurrences) contain only one occurrence. However, it may differ significantly when many occurrences are aggregated on sites. If so, TGB will be biased by a varying prospection intensity between sites and varying TG density, while TGOB may be biased only by the latter factor. In this context, the strengths of TGOB would be leveraged by the search for a criterion to select the best Target-Group of species, which guarantees a low variation of the cumulated TG species density in the environment. The difficulty is that such criterion must be computable from the sets of occurrences of species eligible for the Target-Group. This is an open problem and an area for future work, leading to a clear and reliable background points selection method applicable by SDMs end users.

## Supporting information

S1 Appendix Texts and mathematical proofs. (PDF)

S1 Fig Illustrations of *μ*_*x*_, *f* and *s*_*x*_ along *x* values. An example species density with the standard normal distribution (red curve), the density derived from *μ*_*x*_ chosen uniform over [−5, 5] for the simulation study (black curve), and the observation density *s*_*x*_ of type *LIN* (gold curve).
(PNG)

S2 Fig Illustrations of all simulation results for UB. Plotted true species density (*f*), observation density (*s*_*x*_), observed points density (*fs*_*x*_) and UB estimate of species density in the environmental space. Each situation of the simulation study is represented.
(PNG)

S3 Fig Illustrations of all simulation results for TGOB with FLAT TG species density. Plotted true species density (*f*), observation density (*s*_*x*_), **flat** Target Group species density (*a*), ratio density of species over target group (*f*/*a*) and TGOB estimate of species density in the environmental space. Each situation of the simulation is represented.
(PNG)

S4 Fig Illustrations of all simulation results for TGOB with THICK TG species density. Plotted true species density (*f*), observation density (*s*_*x*_), **thick** Target Group species density (*a*), ratio density of species over target group (*f*/*a*) and TGOB estimate of species density in the environmental space. Each situation of the simulation is represented.
(PNG)

S5 Fig Illustrations of all simulation results for TGOB with THIN TG species density. Plotted true species density (λ_0_), observation density (*s*_*x*_), **thin** Target Group species density (*a*), ratio density of species over target group (λ_0_/*a*) and TGOB estimate of species density in the environmental space. Each situation of the simulation is represented.
(PNG)
