# Supplementary material for: Bias in presence-only niche models related to sampling effort and species niches: Lessons for background point selection
Source: PLoS One. 2020 May 20;15(5):e0232078. doi: 10.1371/journal.pone.0232078 (PMC7239389; doi:10.1371/journal.pone.0232078)
Supplement: S1 Appendix — (PDF) [file pone.0232078.s001.pdf]

S1 Appendix. Texts and mathematical proofs of Manuscript: "Bias in presence-only niche models related to sampling effort and species niches: lessons for background point selection"

Christophe Botella * [1,2,3,5], Alexis Joly[1], Pascal Monestiez[5], Pierre Bonnet[3,4], and François Munoz[6]

[1]INRIA Sophia-Antipolis - ZENITH team, LIRMM - UMR 5506 - CC 477, 161 rue Ada, 34095 Montpellier Cedex 5, France.

[2]INRA, UMR AMAP, F-34398 Montpellier, France.

[3]AMAP, Univ Montpellier, CIRAD, CNRS, INRA, IRD, Montpellier, France.

[4]CIRAD, UMR AMAP, F-34398 Montpellier, France.

[5]BioSP, INRA, Site Agroparc, 84914 Avignon, France.

[6]Université Grenoble Alpes, Laboratoire d'Ecologie Alpine, CS 40700, 38058 Grenoble cedex 9621, France.

*christophe.botella@cirad.fr

1 Text A: Poisson process induced on the environmental domain and factorization of its intensity.

In this part we show how the Poisson process modeling the distribution of observed points in the geographic space, explained in section 2.3 of the manuscript, induce a Poisson point process into the environmental space $Im(x)$ whose intensity function, namely the expected points count per unit of space for a given environment, factorizes to the product of the species intensity function λ and the observation intensity named $\bar{s}$, both defined over $Im(x)$. We justify the interest of looking at bias in the environmental space. Besides, we justify several important hypothesis made in section **2**, such as the almost everywhere continuity of x over D, the almost everywhere continuity of λ over $\mathbb{R}$ and the assumption that it is bounded on any bounded subset of $\mathbb{R}$.

Poisson process induced in the environmental domain. We show hereafter that Z_r follows a general Poisson process [Chiu et al., 2013, Haenggi, 2013] of intensity measure $\Lambda : \mathscr{L}(\mathbb{R}) \rightarrow \mathbb{R}^+, W \rightarrow \int_{x^{-1}(W)} s\lambda \circ x d\mu$, i.e. (i) for any $W \in \mathscr{L}(\mathbb{R})$, $|Z_r \cup x^{-1}(W)| \sim \mathscr{P}(\Lambda(W))$, and (ii) $\forall W_1, W_2 \in \mathscr{L}(\mathbb{R})$ such that $W_1 \cap W_2 = \emptyset$, $|Z_r \cup x^{-1}(W_1)|$ and $|Z_r \cup x^{-1}(W_2)|$ are independent random variables.

First, (i) is straightforward. Let $W \in \mathscr{L}(\mathbb{R})$, then by definition $|Z_r \cup x^{-1}(W)| \sim \mathscr{P}(\int_{x^{-1}(W)} s\lambda \circ x d\mu)$ because Z_r follows a Poisson process over D of intensity measure $s\lambda \circ x$, which is indeed a measure over $\mathscr{L}(\mathbb{R})$ because it is positive by definition, and it is finite because λ is bounded on any bounded subset of $\mathbb{R}$ and $s \in [0,1]$ by definition.

Secondly, (ii) is also straightforward. Let $W_1, W_2 \in \mathscr{L}(\mathbb{R})$ such that $W_1 \cap W_2 = \emptyset$, then $x^{-1}(W_1) \cap x^{-1}(W_2) = \emptyset$ (no spatial point has two different values of x), then $\forall n_1, n_2 \in \mathbb{N}^2, p(|Z_r \cup x^{-1}(W_1)| = n_1, |Z_r \cup x^{-1}(W_2)| = n_2) = p(|Z_r \cup x^{-1}(W_1)| = n_1) p(|Z_r \cup x^{-1}(W_2)| = n_2)$ because Z_r follows a Poisson process over D.

Remark: The Poisson process in the environmental space is equivalent to the one in D if and only x achieves a bijection, or a one-to-one correspondance, between D and the environmental space, which is not the case here as $\mathbb{R}$ is only one dimensional.

Intensity in the environmental domain. We now show that the intensity measure Λ can also be written, for any $W \in \mathscr{L}(\mathbb{R}), \Lambda(W) = \int_W \lambda \bar{s} d\mu_x$ where $\lambda \bar{s}$ is the intensity function of the induced Poisson process over the environmental space $\mathbb{R}$ relatively to the measure μ_x (which is null outside of $Im(x)$) and $\bar{s}$ is defined by:

$$\forall w \in \mathbb{R}, \bar{s}(w) = \begin{cases} \lim\limits_{\delta \to 0} \dfrac{\int_{x^{-1}([w-\frac{\delta}{2}, w+\frac{\delta}{2}])} s d\mu}{\mu_x([w-\frac{\delta}{2}, w+\frac{\delta}{2}])} & \text{if } w \in Im(x) \\ 0 & \text{otherwise, by convention.} \end{cases} \quad (1)$$

Firstly, we show the case where $w \in \mathbb{R} \backslash Im(x)$. We have that $\Lambda(\mathbb{R}) = \Lambda(Im(x))$. This is because $\mu(x^{-1}(\mathbb{R} \backslash Im(x))) = \mu(\{z \in D, x(z) \notin Im(x)\}) = \mu(\{z \in D, x \text{ not continuous at } z\}) = 0$ because x is continuous almost everywhere on D. It implies that any Lebesgue integral computed relatively to μ (Lebesgue measure on $\mathbb{R}^2$) over $x^{-1}(\mathbb{R} \backslash Im(x))$ also equals 0. Thus we could define any value for $\bar{s}$ outside of $Im(x)$, we set it to 0 by convention (which means no observation intensity outside the geographic domain under study).

It remains to show that the writing of $\bar{s}$ is legitimate on $Im(x)$. $Im(x)$, as any subset of $\mathbb{R}$, is a union of intervals and singletons. However, the singletons of $Im(x)$ have an important particularity, they are all atoms of μ_x. More precisely, any singleton w in the connected components of $Im(x)$ is necessarily an atom for the measure μ_x, i.e. $\mu_x(w) > 0$. Indeed, there exists an open subset of the geographic domain $O \subset D$ where x is continuous and reaches the value w somewhere in O. Then, $x(O)$ is an element of an interval of $Im(x)$ that contains w, but as w is not included in any continuous interval of $Im(x)$, this interval is necessarily the singleton $\{w\}$, which implies that $\forall z \in 0, x(z) = w$. Consequently, $\mu_x(w) = \mu(x^{-1}(w)) \geq \mu(O) > 0$ because O is an open subset of $\mathbb{R}^2$ and by definition of the Lebesgue measure on $\mathbb{R}^2$. We have shown that if $w \in Im(x)$ is a singleton of $Im(x)$, it is an atom for μ_x. We can then write $\Lambda(w) = \int_{x^{-1}(w)} s\lambda \circ x\mu = \lambda(w) \int_{x^{-1}(w)} s\mu = \lambda(w) \frac{\int_{x^{-1}(w)} sd\mu}{\mu_x(w)} \mu_x(w)$ $= \lambda(w) \left[\lim_{\delta \to 0} \frac{\int_{x^{-1}([w-\frac{\delta}{2}, w+\frac{\delta}{2}])} sd\mu}{\mu_x([w-\frac{\delta}{2}, w+\frac{\delta}{2}])} \right] \mu_x(w)$. Thus, the definition of $\bar{s}$ in equation 1 holds for singletons of $Im(x)$.

It remains to show that 1 also holds for any non-singleton interval $W \subset Im(x)$. Let $W \subset Im(x)$ be a non-singleton interval. We define the sequence $(C_j := \{C_j^1, ..., C_j^j\})_{j \in \mathbb{N}^*}$ of finite partitions of $[\inf W, \sup W[$. We define it with $\forall j \geq 1, i \leq j$, $C_j^i = [\inf W + (i-1)(\sup W - \inf W)/j, \inf W + i(\sup W - \inf W)/j[$. Then, we note $I_j(W) := \sum_{i=1}^{j} \int_{x^{-1}(C_j^i)} s\lambda \circ x d\mu$ where we can see that $\forall j, I_j(W) = \int_{x^{-1}(W)} s\lambda \circ x d\mu$. Besides,

$$
\begin{aligned}
\lim_{j \to \infty} I_j(W) &= \lim_{j \to \infty} \sum_{i=1}^{j} \lambda(\inf W + i(\sup W - \inf W)/j) \int_{x^{-1}(C_j^i)} sd\mu && (\lambda \text{continuous almost everywhere}) \\
&= \lim_{j \to \infty} \sum_{i=1}^{j} \lambda(\inf W + i(\sup W - \inf W)/j) \frac{\int_{x^{-1}(C_j^i)} sd\mu}{\mu_x(C_j^i)} \mu_x(C_j^i) && (x \text{ continuous on } x^{-1}(C_j^i) \Rightarrow \mu_x(C_j^i) > 0) \\
&= \int_W \lambda \bar{s} d\mu_x
\end{aligned}
$$

Where $\forall w \in W$:

$$
\bar{s}(w) = \lim_{\delta \to 0} \frac{\int_{x^{-1}([w-\frac{\delta}{2}, w+\frac{\delta}{2}])} sd\mu}{\mu_x([w-\frac{\delta}{2}, w+\frac{\delta}{2}])}
$$

Finally, we have shown that for $\forall W \in \mathscr{L}(\mathbb{R}), \Lambda(W) = \int_W \lambda \bar{s} d\mu_x$ where $\lambda \bar{s}$ is the intensity function of the induced Poisson process over the environmental space. We see that this intensity, representing the expected the number of points per unit of space corresponding to a given environment

value, factorizes into a species intensity and $\bar{s}$ that we call observation intensity which depends on the sampling effort s and the environmental variable x, as defined in equation 1. Roughly speaking, $\bar{s}(w)$ is the average of the sampling effort function s over the limit subspace $x^{-1}(w) \subset D$.

Why do we analyse bias in the environmental domain. If s is heterogeneous in space, we may encounter a bias when estimating θ_0 from Z_r, but there is no direct link between the spatial form of s and the bias. Indeed, our target f is a function of x values. So even if s is distributed heterogeneously in D, its variations could cancel in $Im(x)$ and entail no difference on the density of species observed points on $Im(x)$ compared to a uniform sampling on D. That is why it is more relevant to look at the distribution of s over $Im(x)$.

Environmental variable continuity assumption The assumption of almost everywhere continuity of x over D, which means that $\mu(\{z \in D / x \text{ is discontinuous at } z\}) = 0$, is necessary to ensure that $\bar{s}$ and s_x are well defined on $Im(x)$. Let's recall that $Im(x) = \{w \in \mathbb{R} / \exists z \in D / x(z) = w \text{ and } x \text{ is continuous at } z\}$ is the set of values for which there exist fibers of x in D at which x is continuous. The almost everywhere continuity allows discontinuities of x over negligible areas of D, basically points and lines, which is useful because it allows x is a rasterized environmental variable, a continuously varying variable, or even a mixture of both.

Species intensity continuity assumption λ which is continuous almost everywhere over $Im(x)$. This hypothesis is useful to allow this function to be not continuous on certain points. For instance, Maxent [Phillips and Dudík, 2008] uses threshold functions in its model. Besides, this hypothesis doesn't seem limiting, because it is hard to imagine a species density function that would have discontinuity points over an infinite and non-countable number of points, even if such function can be theoretically built.

2 Text B: Modeling the species niche with a gaussian density

Here we describe our choice of gaussian density for f in simulation. Of course, we cannot cover the huge variety of niche models, so we chose to illustrate classic ecological types. We assume that the realized niche of a species corresponds to its fundamental niche, in the sense of Hutchinson [1957]. The expected species abundance only depends on the suitability of environment described by x. Even if the spatial variation of the abiotic environment is known to be a strong determinant of species distribution, it is not the only factor affecting it, there is also the spatial dispersal constraints and the interactions with other organisms (Pulliam [2000],Soberón [2007]). Species distribution along environmental gradients are often thought to be unimodal and tappered, and the more precise choice of modeling the species density as a gaussian function along environmental gradient is quite

comon in ecology (Franklin [2010]). The maximum of f is called the **optimum**, and the inverse of its variance, its **specialization**. Indeed, those quantities are of main interest for ecological applications, and it is crucial to study their biases. Chosing the gaussian density for f can be interpreted as setting the constraints that the expected x of a given species individual is μ_0 ($\int_{\mathbb{R}} f(w)wdw = \mu_0$, optimum constraint), the variance of x over many individuals is σ_0^2 ($\int_{\mathbb{R}} f(w)(w-\mu_0)^2 dw = \sigma_0^2$, specialization constraint), and f is of maximum entropy.

3 Text C: Fitting the UB model to data

We here present the details of the UB fitting method, as described in Berman and Turner [1992] and Renner et al. [2015]. The UB method is fitted by maximizing the log-likelihood of the Poisson point process model of intensity λ_θ, defined over the domain D, with observed species occurrences $Z = \{z_1, ..., z_n\}$, with respect to the model parameters $\theta := (\alpha, \beta_1, \beta_2)$:

$$\begin{aligned}\mathscr{L}(z_1,...,z_n|\theta) &= \log(p(z_1,...,z_n|\theta)) \\ &= \log\left(e^{-\int_D \lambda_\theta \circ x d\mu} \prod_{i=1}^{n} \lambda_\theta(x(z_i))\right) \\ &= \sum_{i=1}^{n} \log(\lambda_\theta(x(z))) - \int_D \lambda_\theta \circ x d\mu\end{aligned}$$

In general, the integral term cannot be computed exactly. We rather use a numerical approximation. The integral is replaced by a weighted sum of λ_θ computed at some background/quadrature points, $Z^q = \{z_1^q, ..., z_Q^q\}$ where Q is the number of background points. In MAXENT literature, quadrature points are often called pseudo-absences. Berman and Turner [1992] re-express the likelihood by including $z_1, ..., z_n$ among background points, and defining samples weights. It gives a classic Poisson regression likelihood:

$$\begin{aligned}\mathscr{L}(z_1,...,z_n|\theta) &\approx \sum_{j=1}^{Q} 1_{z\in Z} \log(\lambda_\theta(x(z_j^q))) - w_j \lambda_\theta(x(z_j^q)) \\ &= \sum_{j=1}^{Q} w_j \left(y_j \log(\lambda_\theta(x(z_j^q))) - \lambda_\theta(x(z_j^q))\right)\end{aligned}$$

Where the y_j correspond to the Poisson regression counts (called pseudo-counts because they can be non integers), and w_j the samples weights. We define **the background points** $Z^q \backslash Z$ and their weights so that $\sum_{i=1}^{n} w_i \lambda_\theta(x(z_i^q)) \approx \int_D \lambda_\theta \circ x d\mu$. A unbiased and popular manner to approximate the integral is the Monte Carlo method, which uses the average over uniformly sampled points on D to approximate the integral. However, we must prevent $z_1, ..., z_n$ from biasing our approximation, because they are not uniformly distributed in D. We give them a total weight in the sum that is negligible compared to the background points drawn uniformly :

$$\forall j \in [|1, Q|],\ w_j \begin{cases} = \frac{\mu(D)}{100n} & \text{if } z_j^q \in Z \\ = \frac{99\mu(D)}{100(Q-n)} & \text{otherwise} \end{cases}$$

With this setting, all weights sum to $\mu(D)$, while weights of species reported points alone represent only 1% of this value. This way, the approximation of $\int_D \lambda_\theta \circ x d\mu$ with background

points is not affected by reported points. Now, the standard formulation of the Poisson parameter in the Poisson Generalized Linear Model is slighlty different from our model. It doesn't use our parametrization of λ_θ with the gaussian distribution parameters $\theta = (K, \mu, \sigma^2)$, but another equivalent parametrization. We note this equivalent function λ'_γ, called the log-linear predictor, for any z like this : $\lambda_\theta(x(z)) = \lambda'_\gamma(x(z)) = \exp\left(\alpha + \beta_1 x(z) + \beta_2 x(z)^2\right)$ where $\gamma = (\alpha, \beta_1, \beta_2)$ are the parameters of the log-linear predictor that are returned by standard Generalized Linear Model softwares. We can now easily recover our parameters of interest μ and σ by identification:

$$\begin{aligned} \forall z \in D,\ \lambda_\theta(x(z)) &= \exp\left(K - \tfrac{(x(z)-\mu)^2}{2\sigma^2}\right) \\ &= \exp\left(\alpha + \beta_1 x(z) + \beta_2 x(z)^2\right) \quad \text{with} \left\{ \begin{array}{lcl} \beta_1 &=& \frac{\mu_0}{\sigma_0^2} \\ \beta_2 &=& \frac{-1}{2\sigma_0^2} \end{array} \right. \Leftrightarrow \left\{ \begin{array}{lcl} \mu_0 &=& \frac{\beta_1}{2\beta_2} \\ \sigma_0 &=& \frac{1}{\sqrt{-2\beta_2}} \end{array} \right. \end{aligned}$$

Where β_2 is strictly negative. We can now compute the Generalized Linear Model (with R package `glm`) to estimate parameter values β_1, β_2 that maximize the likelihood, for given y_js, $x(z_j^q)$s and w_js.

4 Text D: Proof of asymptotic UB estimate (Equation 2)

This part proves equation **2** (section 4.1 in manuscript) which expresses the expected UB estimate as the minimzer of a divergence to the observed species density fs_x. We are interested in the asymptotical estimate of the environmental density of the UB method given that the observed points follow the Poisson process: $IPP(s\lambda \circ x)$. Our target is the intensity function $\lambda(x(.)))$ but we can only estimate it, at best, up to a constant factor as it is multiplied by s, of unknown global scale, in the generating process as already shown in Fithian and Hastie [2013] and Hastie and Fithian [2013]. We may still estimate the relative intensity function by maximizing the joint likelihood of points position, conditional to the number of points generated by the process. For a finite sample $z_1, ..., z_n \in D$ of point realizations of the process, it is written:

$$p(z_1, ..., z_n | n, \theta) = \prod_{i=1}^{n} \frac{\lambda_\theta(x(z_i))}{\int_D \lambda_\theta \circ x d\mu}$$

Thus, the maximum likelihood parameter estimate of the intensity function is

$$\hat{\theta}_{UB} = \underset{\theta}{\text{argmax}}\ P(z_1, ..., z_n | n, \theta) = \underset{\theta}{\text{argmin}}\ -\frac{1}{n}\log(P(z_1, ..., z_n | n, \theta))$$

We recall that $\bar{s}$, λ and λ_θ are continuous μ_x-almost everywhere. Then, the limit of the above averaged negative Log likelihood when $n \to +\infty$ can be rewritten as follows:

$$
\begin{aligned}
& \lim_{n\to\infty} -\frac{1}{n}\sum_{i=1}^{n} \log\left(\frac{\lambda_\theta(x(z_i))}{\int_D \lambda_\theta \circ x d\mu}\right) \\
= \; & \mathbb{E}\left(-\log\left(\frac{\lambda_\theta(x(z_1))}{\int_D \lambda_\theta \circ x d\mu}\right)\right) \\
= \; & -\int_D \frac{s\lambda \circ x}{\int_D s\lambda \circ x d\mu} \log\left(\frac{\lambda_\theta \circ x}{\int_D \lambda_\theta \circ x d\mu}\right) d\mu \\
= \; & -\lim_{\delta\to 0^+} \sum_{k=0}^{N(\delta)} \int_D 1_{z\in x^{-1}([a_k,a_k+\delta])} \frac{s(z)\lambda(x(z))}{\int_D s\lambda \circ x d\mu} \log\left(\frac{\lambda_\theta(x(z))}{\int_D \lambda_\theta \circ x d\mu}\right) dz \\
= \; & -\lim_{\delta\to 0^+} \sum_{k=0}^{N(\delta)} \frac{\lambda(a_k)}{\int_D s\lambda \circ x d\mu} \log\left(\frac{\lambda_\theta(a_k)}{\int_D \lambda_\theta \circ x d\mu}\right) \int_{x^{-1}([a_k,a_k+\delta])} s d\mu \qquad (x(z)\to a_k \text{ and } \lambda,\lambda_\theta \text{ continuous } \mu_x\text{-almost everywhere}) \\
= \; & -\lim_{\delta\to 0^+} \sum_{k=0}^{N(\delta)} \left[\frac{\lambda(a_k)}{\int_D s\lambda \circ x d\mu} \log\left(\frac{\lambda_\theta(a_k)}{\int_D \lambda_\theta \circ x d\mu}\right) \right. \\
& \left. \frac{\int_{x^{-1}([a_k,a_k+\delta])} s d\mu}{\mu_x([a_k,a_k+\delta])} \mu_x([a_k,a_k+\delta])\right] \\
= \; & -\int_{\mathbb{R}} \frac{\bar{s}\lambda}{\int_D s\lambda \circ x d\mu} \log\left(\frac{\lambda_\theta}{\int_D \lambda_\theta \circ x d\mu}\right) d\mu_x \\
\alpha \; & -\int_{\mathbb{R}} \frac{s_x f}{\int_{\mathbb{R}} s_x f d\mu_x} \log\left(\frac{\lambda_\theta}{\int_D \lambda_\theta \circ x d\mu}\right) d\mu_x \qquad (\text{factor } > 0 \text{ and independent of } \theta) \\
= \; & -\int_{\mathbb{R}} \frac{s_x f}{\int_{\mathbb{R}} s_x f d\mu_x} \log\left(\frac{\lambda_\theta}{\int_{Im(x)} \lambda_\theta d\mu_x}\right) d\mu_x \qquad (\text{same method}) \\
= \; & \int_{\mathbb{R}} \frac{s_x f}{\int_{\mathbb{R}} s_x f d\mu_x} \left[\log\left(\frac{s_x f}{\int_{\mathbb{R}} s_x f d\mu_x}\right) - \log(f_\theta)\right] d\mu_x \qquad (\text{adding term independent of } \theta) \\
= \; & \mathscr{D}_{KL}^{\mu_x}(f s_x || f_\theta)
\end{aligned}
$$

Where $a_k = inf(Im(x)) + k\delta$ and $N(\delta)$ is the quotient of the euclidean division of $|Im(x)|$ by δ. This way, we have $[a_0, a_{N(\delta)}] \subset Im(x) \subset [a_0, a_{N(\delta)} + \delta]$.

5 Text E: μ_x weighted KL-Divergence

The divergence is weighted by μ_x, the measure of the spatial area associated with any x value. It means that on parts of $Im(x)$ where $\mu_x = 0$ (*i.e.* environment not in D or of negligible area), $f_{\hat{\theta}_{UB}}$ is unconstrained, so it is allowed to take any shape, and it will depend on estimated parameters. As a consequence, the prediction of species intensity outside the enviromental range covered in D will be highly influenced by a misspecification of distribution model. This remark is also true for the following methods. The μ_x weighting will also lead approximation error compromises when $f_{\hat{\theta}_{UB}}$

cannot fit exactly to $f \circ s_x$. For example, if the parametrization of f_θ doesn't allow it to fit well to $s_x f$ over both subset $W_1, W_2 \subset Im(x)$ with $W_1 \cap W_2 = \emptyset, |W_1| = |W_2|$, and $\mu_x(W_1) > \mu_x(W_2)$, then the estimate should fit better on W_1 than on W_2.

6 Text F: A sample from the sampling effort proportional density as background.

In this part we demonstrate the optimality of the theoritical method consisting of using an large sample of background points directly drawn independantly from the sampling effort proportional density over D, which is the method **ApproxFactorBiasOut** introduced in Dudík et al. [2006].

We now assume that we have a sample $z_1^s, ..., z_{n_0}^s$ from $s / \int_D s(z)dz$, *i.e.* points distributed according to the proportional density of the sampling effort. We use these points as equally weighted background points in the Poisson process likelihood. We re-express the asymptotic estimator associated with this procedure. Like previously, we write the limit of the averaged negative log-likelihood, with now both n and n_0 tend to infinity:

$$
\begin{aligned}
& \lim_{\substack{n\to\infty \\ n_0\to\infty}} -\frac{1}{n}\sum_{i=1}^{n} \log\left(\frac{\lambda_\theta(x(z_i))}{\sum_{j=1}^{n_0} \frac{\mu(D)}{n_0}\lambda_\theta(x(z_j^s))}\right) \\
= \; & \lim_{n\to\infty} -\frac{1}{n}\textstyle\sum_{i=1}^{n} \log\left(\frac{\lambda_\theta(x(z_i))}{\int_D \frac{s}{\int_D s d\mu}\lambda_\theta\circ x \, d\mu}\right) \\
= \; & -\int_D \frac{s\lambda\circ x}{\int_D s\,\lambda\circ x\, d\mu} \log\left(\frac{\lambda_\theta\circ x}{\int_D \frac{s}{\int_D s d\mu}\lambda_\theta\circ x\, d\mu}\right) d\mu \\
= \; & -\int_{\mathbb{R}} \frac{s_x f}{\int_{\mathbb{R}} s_x f d\mu_x} \log\left(\frac{\lambda_\theta(w)}{\int_D \frac{s}{\int_D s d\mu}\lambda_\theta\circ x\, d\mu}\right) d\mu_x \qquad \text{(previous method, factor independent of } \theta\text{)} \\
= \; & -\int_{\mathbb{R}} \frac{s_x f}{\int_{\mathbb{R}} s_x f d\mu_x} \log\left(\frac{\lambda_\theta}{\int_{\mathbb{R}} f_\theta \frac{\bar{s}}{\int_D s d\mu} d\mu_x}\right) d\mu_x \qquad \text{(previous method)} \\
= \; & \int_{\mathbb{R}} \frac{s_x f}{\int_{\mathbb{R}} s_x f d\mu_x} \log\left(\frac{f s_x}{f_\theta s_x}\right) d\mu_x \qquad \text{(adding constant term and neg-entropy of } f s_x\text{)} \\
= \; & \mathscr{D}_{KL}^{\mu_x}(f s_x || f_\theta s_x)
\end{aligned}
$$

$$
\mathbb{E}(\hat{\theta}_{AFBO}) = \underset{\theta}{\text{Argmin}}\; \mathscr{D}_{KL}^{\mu_x}(f s_x || f_\theta s_x)
$$

Thus, $f_{\hat{\theta}_{AFBO}}$ will converge to f, except on parts where $s_x \mu_x = 0$ and the method gives an unbiased estimate of the species niche.

7 Text G: Proof of asymptotic TGOB estimate (Equation 5)

This part proves equation **5** (section 4.7 in manuscript) which expresses the expected TGOB estimate as the minimzer of a divergence from $f_\theta a$ to f, which means it fits to f/a. For this part we assume, on top of previous conditions (Riemann integrability of λ and $\bar{s}$), that a is Riemann inte-

grable on $\mathbb{R}$. We recall that the ensemble of observed points of species from the Target-Group is noted Z^{tg}. On the same principle than previously, we re-express the limit of the averaged negative log likelihood when the background points are drawn according to the TG species density:

$$
\begin{aligned}
& \lim_{\substack{n\to\infty \\ |Z^{tg}|\to\infty}} -\frac{1}{n}\sum_{i=1}^{n} \log\left(\frac{\lambda_\theta(x(z_i))}{\sum\limits_{z\in Z^{tg}} \frac{\mu(D)}{|Z^{tg}|}\lambda_\theta(x(z))}\right) & \\
= \; & \lim_{n\to\infty} -\frac{1}{n}\sum_{i=1}^{n} \log\left(\frac{\lambda_\theta(x(z_i))}{\int_D s\, a\circ x\, \lambda_\theta\circ x\, d\mu}\right) & \text{(TG points drawn from density } s\, a\circ x\text{)} \\
= \; & -\int_D s\, \lambda\circ x \log\left(\frac{\lambda_\theta\circ x}{\int_D s\, a\circ x\, \lambda_\theta\circ x\, d\mu}\right) d\mu & \text{(species points drawn from density } s\, \lambda\circ x\text{)} \\
\alpha \; & -\int_{\mathbb{R}} \frac{s_x f}{\int_{\mathbb{R}} s_x f d\mu_x} \log\left(\frac{\lambda_\theta}{\int_D s\, a\circ x\, \lambda_\theta\circ x\, d\mu}\right) d\mu_x & \text{(previous method, factor independent of } \theta\text{)} \\
= \; & -\int_{\mathbb{R}} \frac{s_x f}{\int_{\mathbb{R}} s_x f d\mu_x} \log\left(\frac{\lambda_\theta}{\int_{\mathbb{R}} \bar{s} a \lambda_\theta d\mu_x}\right) d\mu_x & \text{(previous method)} \\
= \; & -\int_{\mathbb{R}} \frac{s_x f}{\int_{\mathbb{R}} s_x f d\mu_x} \log\left(f_\theta s_x a\right) d\mu_x & \text{(adding term independent of } \theta\text{)} \\
= \; & D_{KL}^{\mu_x}\left(f s_x || f_\theta s_x a\right) & \text{(substrating entropy of } \lambda \bar{s} p_x\text{)}
\end{aligned}
$$

References

Berman, M. and Turner, T. R. (1992). Approximating point process likelihoods with glim. *Applied Statistics*, pages 31–38.

Chiu, S. N., Stoyan, D., Kendall, W. S., and Mecke, J. (2013). *Stochastic geometry and its applications*. John Wiley & Sons.

Dudík, M., Phillips, S. J., and Schapire, R. E. (2006). Correcting sample selection bias in maximum entropy density estimation. In *Advances in neural information processing systems*, pages 323–330.

Fithian, W. and Hastie, T. (2013). Finite-sample equivalence in statistical models for presence-only data. *The annals of applied statistics*, 7(4):1917.

Franklin, J. (2010). *Mapping species distributions: spatial inference and prediction*. Cambridge University Press.

Haenggi, M. (2013). Stochastic geometry for wireless networks, cambridge uni.

Hastie, T. and Fithian, W. (2013). Inference from presence-only data; the ongoing controversy. *Ecography*, 36(8):864–867.

Hutchinson, G. E. (1957). Cold spring harbor symposium on quantitative biology. *Concluding remarks*, 22:415–427.

Phillips, S. J. and Dudík, M. (2008). Modeling of species distributions with maxent: new extensions and a comprehensive evaluation. *Ecography*, 31(2):161–175.

Pulliam, H. R. (2000). On the relationship between niche and distribution. *Ecology letters*, 3(4):349–361.

Renner, I. W., Elith, J., Baddeley, A., Fithian, W., Hastie, T., Phillips, S. J., Popovic, G., and

Warton, D. I. (2015). Point process models for presence-only analysis. *Methods in Ecology and Evolution*, 6(4):366–379.

Soberón, J. (2007). Grinnellian and eltonian niches and geographic distributions of species. *Ecology letters*, 10(12):1115–1123.
