# Supplementary figures and images for: Bias in presence-only niche models related to sampling effort and species niches: Lessons for background point selection
Source: PLoS One. 2020 May 20;15(5):e0232078. doi: 10.1371/journal.pone.0232078 (PMC7239389; doi:10.1371/journal.pone.0232078)

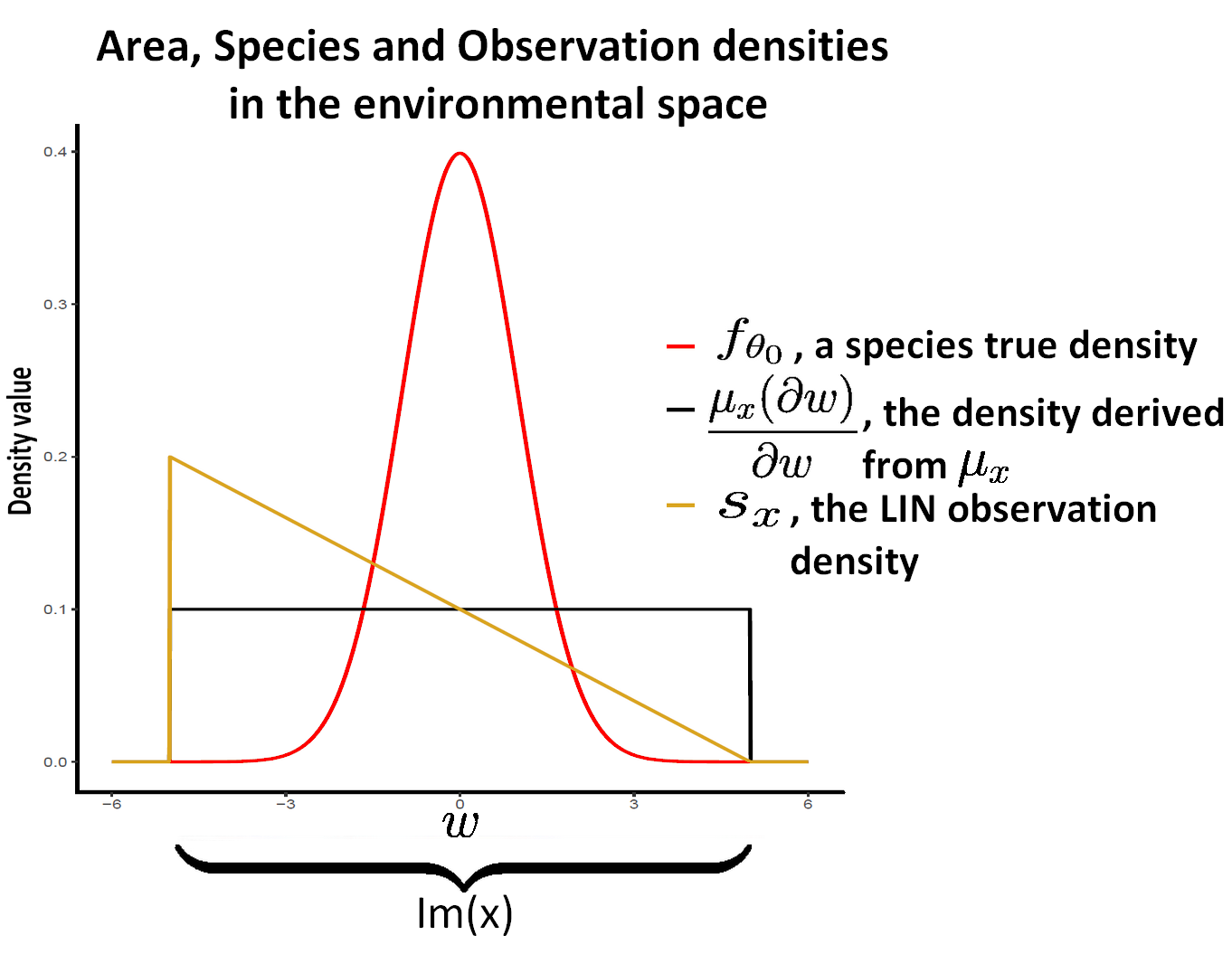

Supplement: S1 Fig — An example species density with the standard normal distribution (red curve), the density derived from μx chosen uniform over [−5, 5] for the simulation study (black curve), and the observation density sx of type LIN (gold curve). (PNG) [file pone.0232078.s002.png]

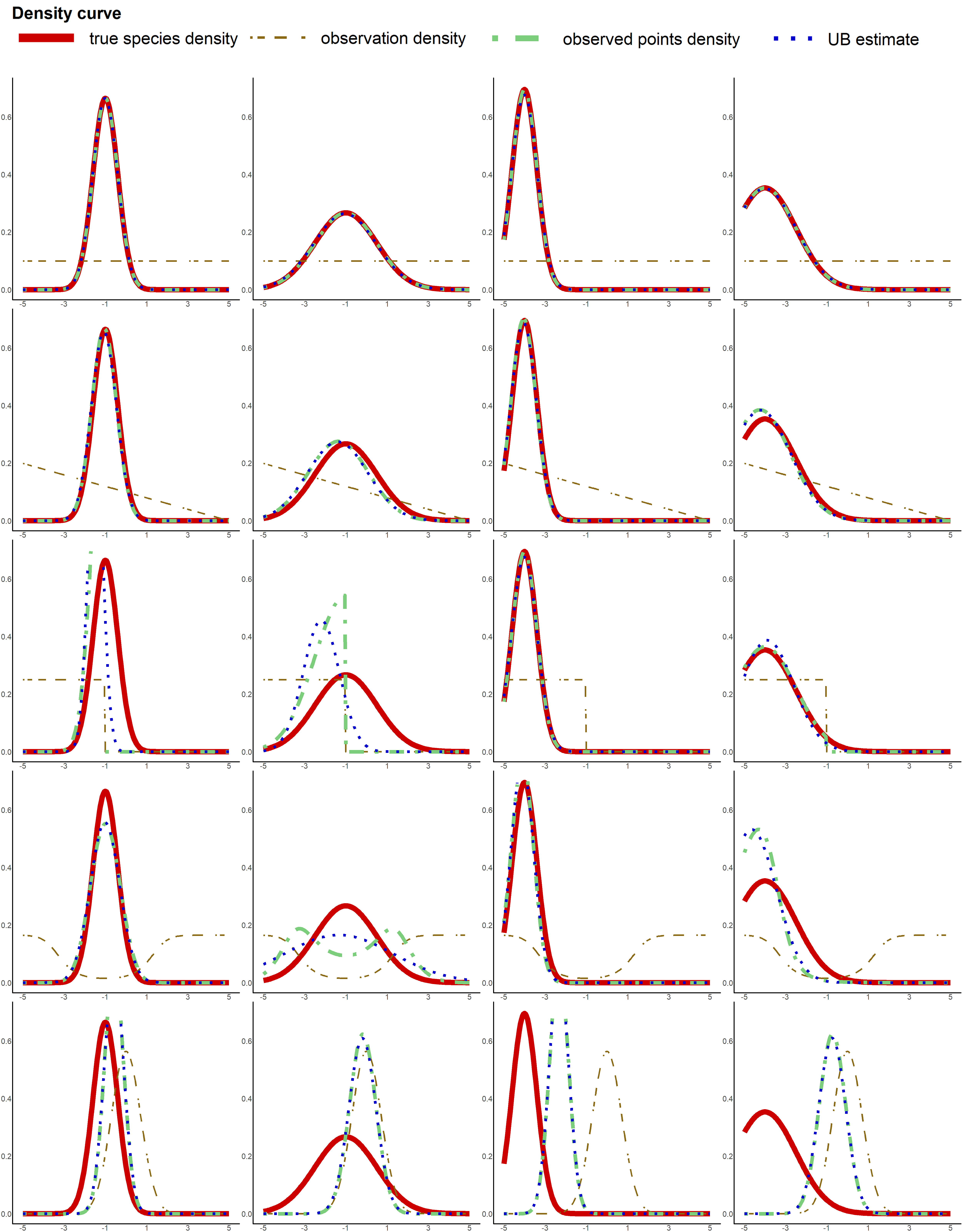

Supplement: S2 Fig — Plotted true species density (f), observation density (sx), observed points density (fsx) and UB estimate of species density in the environmental space. Each situation of the simulation study is represented. (PNG) [file pone.0232078.s003.png]

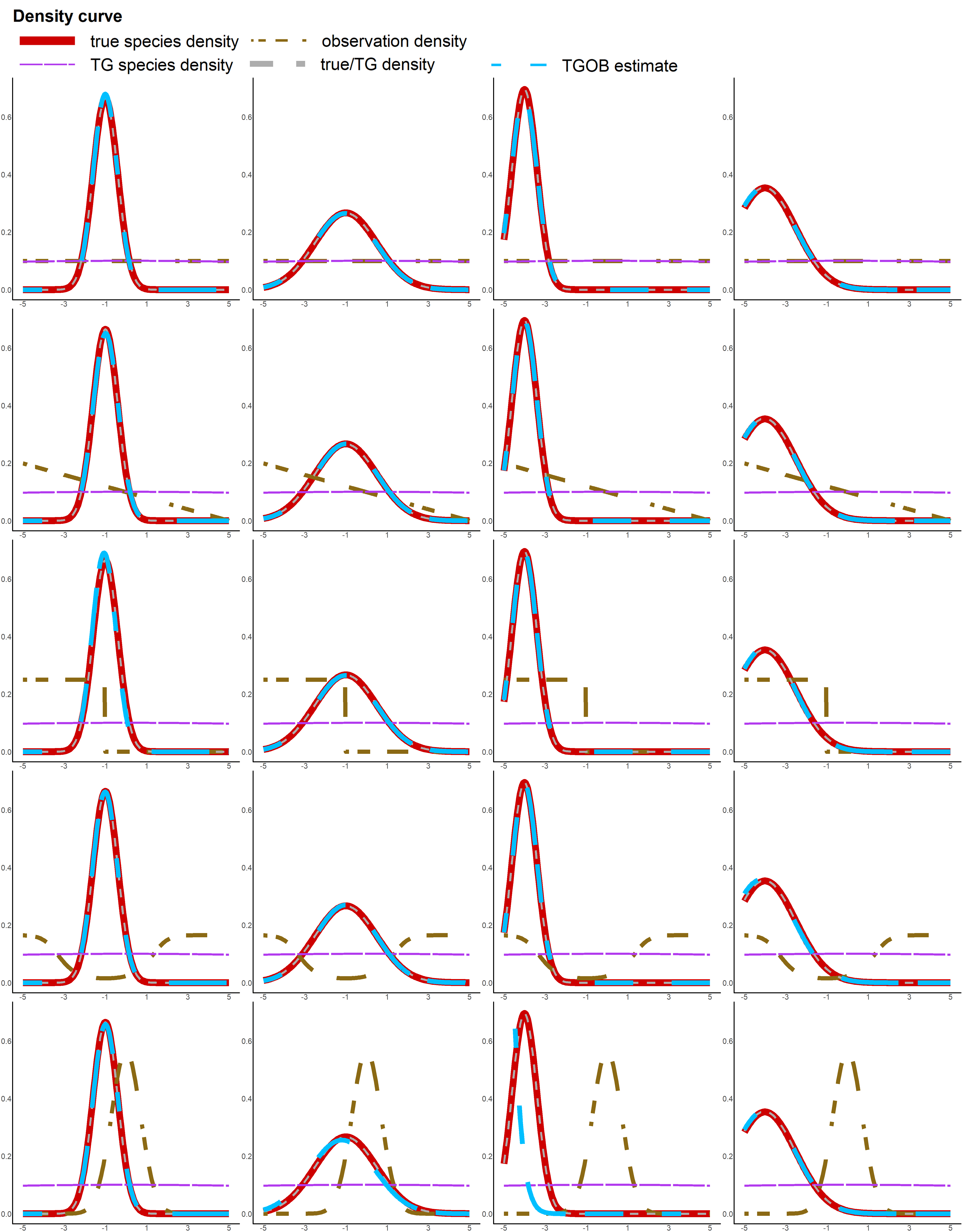

Supplement: S3 Fig — Plotted true species density (f), observation density (sx), flat Target Group species density (a), ratio density of species over target group (f/a) and TGOB estimate of species density in the environmental space. Each situation of the simulation is represented. (PNG) [file pone.0232078.s004.png]

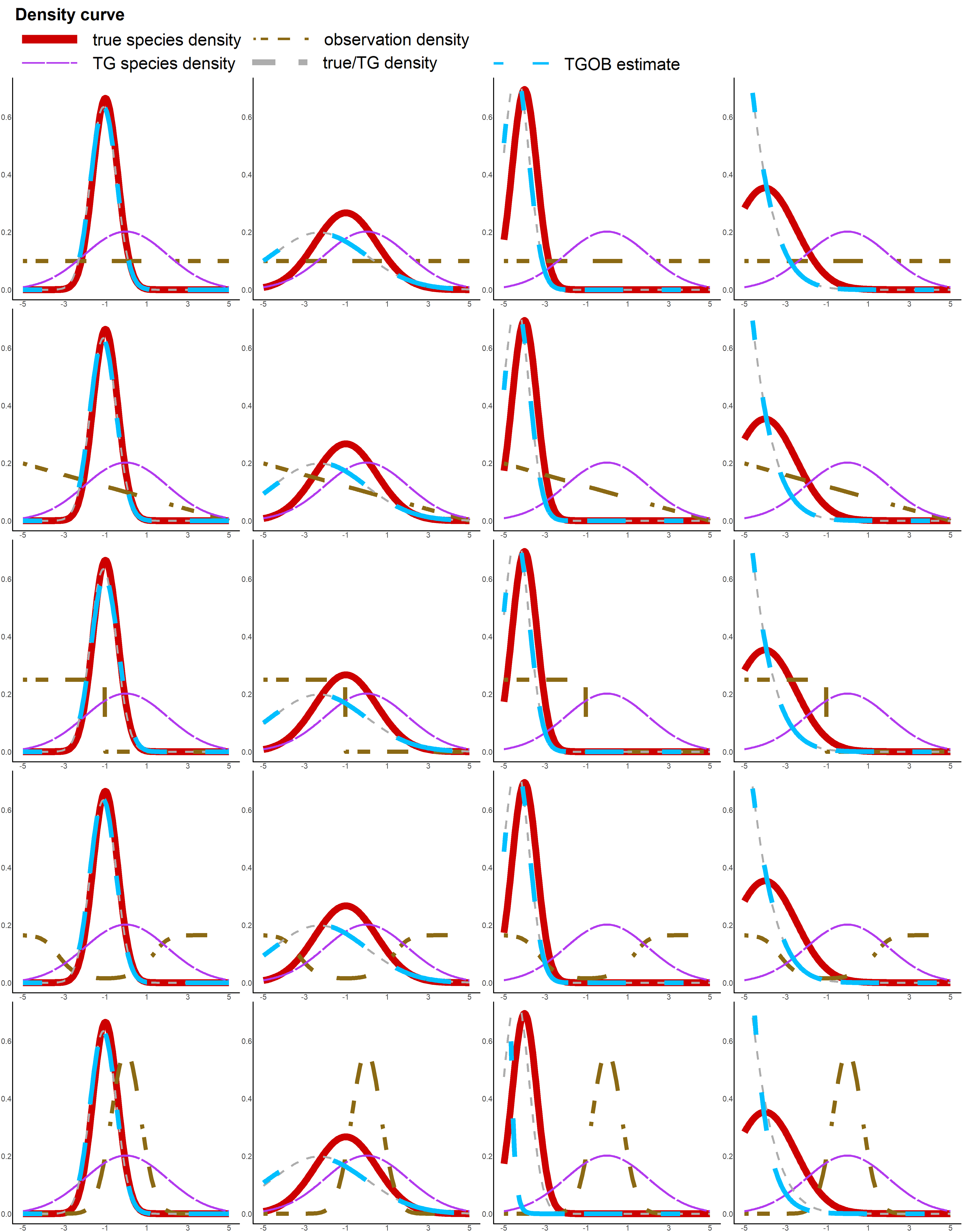

Supplement: S4 Fig — Plotted true species density (f), observation density (sx), thick Target Group species density (a), ratio density of species over target group (f/a) and TGOB estimate of species density in the environmental space. Each situation of the simulation is represented. (PNG) [file pone.0232078.s005.png]

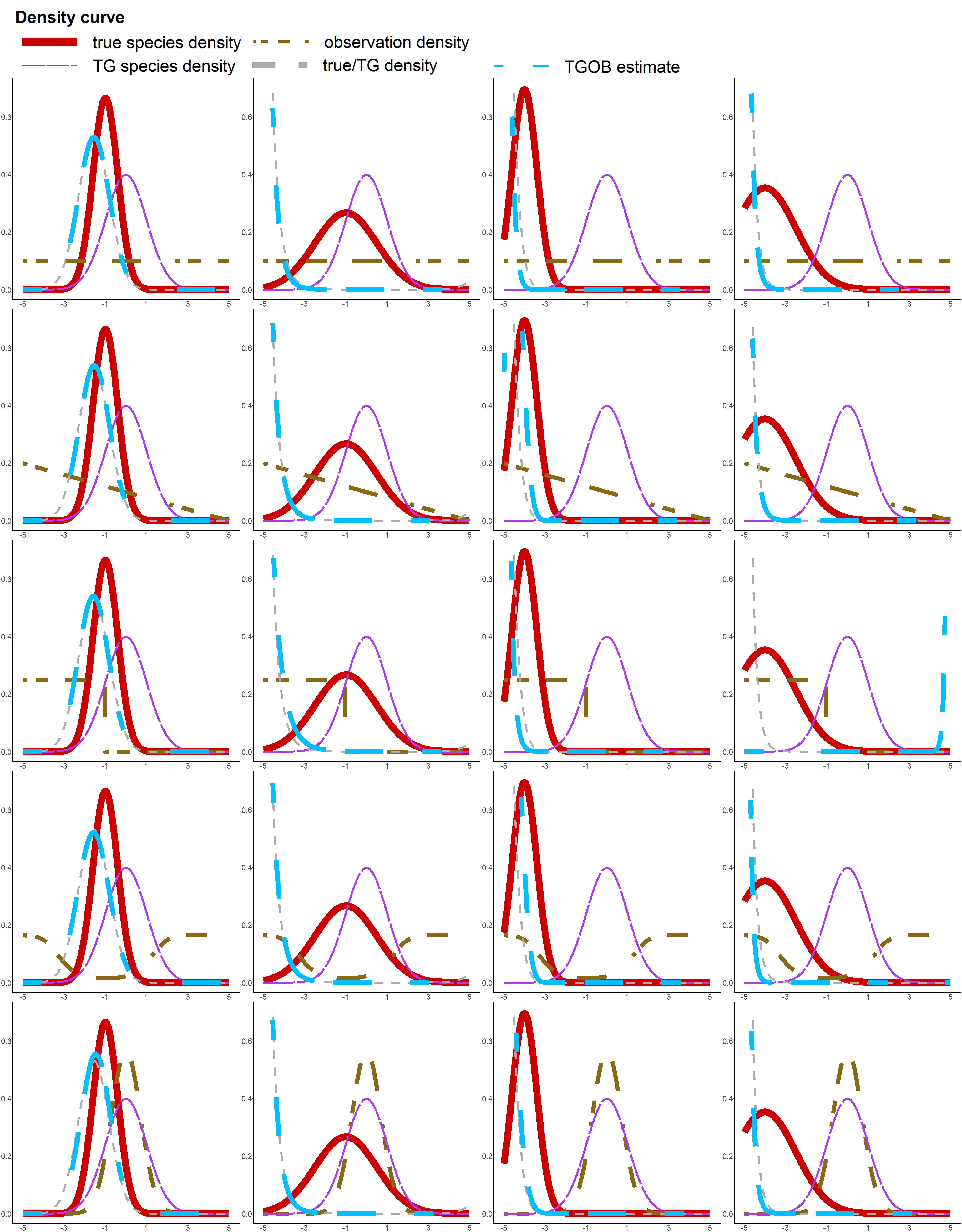

Supplement: S5 Fig — Plotted true species density (λ0), observation density (sx), thin Target Group species density (a), ratio density of species over target group (λ0/a) and TGOB estimate of species density in the environmental space. Each situation of the simulation is represented. (PNG) [file pone.0232078.s006.png]
